# Reproducible grey matter patterns index a multivariate, global alteration of brain structure in schizophrenia and bipolar disorder

**DOI:** 10.1038/s41398-018-0225-4

**Published:** 2019-01-17

**Authors:** Emanuel Schwarz, Nhat Trung Doan, Giulio Pergola, Lars T Westlye, Tobias Kaufmann, Thomas Wolfers, Ralph Brecheisen, Tiziana Quarto, Alex J Ing, Pasquale Di Carlo, Tiril P Gurholt, Robbert L Harms, Quentin Noirhomme, Torgeir Moberget, Ingrid Agartz, Ole A Andreassen, Marcella Bellani, Alessandro Bertolino, Giuseppe Blasi, Paolo Brambilla, Jan K Buitelaar, Simon Cervenka, Lena Flyckt, Sophia Frangou, Barbara Franke, Jeremy Hall, Dirk J Heslenfeld, Peter Kirsch, Andrew M McIntosh, Markus M Nöthen, Andreas Papassotiropoulos, Dominique J-F de Quervain, Marcella Rietschel, Gunter Schumann, Heike Tost, Stephanie H Witt, Mathias Zink, Andreas Meyer-Lindenberg, The IMAGEMEND Consortium, Karolinska Schizophrenia Project (KaSP) Consortium

**Affiliations:** 1 Department of Psychiatry and Psychotherapy, Central Institute of Mental Health, Medical Faculty Mannheim, University of Heidelberg, Mannheim, Germany; 2 Norwegian Centre for Mental Disorders Research (NORMENT), KG Jebsen Centre for Psychosis Research, Division of Mental Health and Addiction, Institute of Clinical Medicine, University of Oslo, Oslo, Norway; 3 Department of Basic Medical Sciences, Neuroscience and Sense Organs, University of Bari Aldo Moro, Bari, Italy; 4 Department of Psychology, University of Oslo, Oslo, Norway; 5 Department of Human Genetics, Radboud University Medical Center, Nijmegen, The Netherlands; 6 Donders Center for Cognitive Neuroimaging, Radboud University, Nijmegen, The Netherlands; 7 Maastricht University Medical Center, Maastricht, The Netherlands; 8 Cognitive Brain Research Unit, Department of Psychology and Logopedics, Faculty of Medicine, University of Helsinki, Helsinki, Finland; 9 Centre for Population Neuroscience and Stratified Medicine (PONS) and MRC-SGDP Centre, Institute of Psychiatry, Psychology & Neuroscience, King’s College London, London, UK; 10 Brain Innovation B.V., Maastricht, The Netherlands; 11 Centre for Psychiatry Research, Department of Clinical Neuroscience, Karolinska Institutet, & Stockholm County Council, Stockholm, Sweden; 12 Department of Psychiatry Research, Diakonhjemmet Hospital, Oslo, Norway; 13 Section of Psychiatry, Azienda Ospedaliera Universitaria Integrata Verona, Verona, VR Italy; 14 Department of Neurosciences, Biomedicine and Movements Sciences, University of Verona, Verona, VR Italy; 15 Institute of Psichiatry, Policlinico Bari, Azienda Ospedaliero Universitaria Consorziale Policlinico Bari, Bari, BA Italy; 16 Azienda Ospedaliero-Universitaria Consorziale Policlinico, Bari, Italy; 17 Department of Neurosciences and Mental Health, Fondazione IRCCS Ca’ Granda Ospedale Maggiore Policlinico, University of Milan, Milan, Italy; 18 Donders Institute for Brain, Cognition and Behaviour, Radboudumc, Nijmegen The Netherlands; 19 Karakter Child and Adolescent Psychiatry University Center, Nijmegen, The Netherlands; 20 Department of Psychiatry, Icahn School of Medicine at Mount Sinai, New York, NY USA; 21 Departments of Human Genetics and Psychiatry, Radboud University Medical Center, Nijmegen, The Netherlands; 22 Neuroscience and Mental Health Research Institute, Cardiff University, Maindy Road, Cardiff, CF24 4HQ UK; 23 Department of Cognitive Psychology, Vrije Universiteit Amsterdam, Amsterdam, The Netherlands; 24 Department of Clinical Psychology, Central Institute of Mental Health, Medical Faculty Mannheim, University of Heidelberg, Heidelberg, Germany; 25 Bernstein Center for Computational Neuroscience Heidelberg-Mannheim, Mannheim, Germany; 26 Division of Psychiatry, University of Edinburgh, Royal Edinburgh Hospital, Edinburgh, EH10 5HF UK; 27 Centre for Cognitive Ageing and Cognitive Epidemiology, University of Edinburgh, George Square, Edinburgh, EH8 9JZ UK; 28 Institute of Human Genetics, University of Bonn, School of Medicine & University Hospital Bonn, Bonn, Germany; 29 Department of Genomics, Life & Brain Center, University of Bonn, Bonn, Germany; 30 Division of Molecular Neuroscience, Department of Psychology, University of Basel, CH-4055 Basel, Switzerland; 31 Transfaculty Research Platform Molecular and Cognitive Neuroscience, University of Basel, Basel, Switzerland; 32 Psychiatric University Clinics, University of Basel, CH-4055 Basel, Switzerland; 33 Department Biozentrum, Life Sciences Training Facility, University of Basel, CH-4056 Basel, Switzerland; 34 Division of Cognitive Neuroscience, Department of Psychology, University of Basel, CH-4055 Basel, Switzerland; 35 Department of Genetic Epidemiology in Psychiatry, Central Institute of Mental Health, Medical Faculty Mannheim, Heidelberg University, Heidelberg, Germany; 36 District Hospital Mittelfranken, Department of Psychiatry, Psychotherapy and Psychosomatics, Ansbach, Germany

## Abstract

Schizophrenia is a severe mental disorder characterized by numerous subtle changes in brain structure and function. Machine learning allows exploring the utility of combining structural and functional brain magnetic resonance imaging (MRI) measures for diagnostic application, but this approach has been hampered by sample size limitations and lack of differential diagnostic data. Here, we performed a multi-site machine learning analysis to explore brain structural patterns of T1 MRI data in 2668 individuals with schizophrenia, bipolar disorder or attention-deficit/ hyperactivity disorder, and healthy controls. We found reproducible changes of structural parameters in schizophrenia that yielded a classification accuracy of up to 76% and provided discrimination from ADHD, through it lacked specificity against bipolar disorder. The observed changes largely indexed distributed grey matter alterations that could be represented through a combination of several global brain-structural parameters. This multi-site machine learning study identified a brain-structural signature that could reproducibly differentiate schizophrenia patients from controls, but lacked specificity against bipolar disorder. While this currently limits the clinical utility of the identified signature, the present study highlights that the underlying alterations index substantial global grey matter changes in psychotic disorders, reflecting the biological similarity of these conditions, and provide a roadmap for future exploration of brain structural alterations in psychiatric patients.

## Introduction

Schizophrenia is a severe neuropsychiatric disorder affecting approximately 0.7% of the population^1^. A large spectrum of experimental approaches has been used to identify neural alterations in schizophrenia^2,3^. Among these, magnetic resonance imaging (MRI) has received particularly strong interest^4^ due to its non-invasiveness, high efficiency in acquiring brain-wide information on structure and function, and the ubiquitous availability of scanners, enabling the accumulation of large sample sizes. Meta-analyses of MRI data have demonstrated the presence of widespread brain-structural changes in patients^5–14^, and machine learning, whereby combined effects of numerous predictors can be exploited, has been used to identify predictive patterns that explain a substantial amount of schizophrenia-associated variation^15,16^.

With a few notable exceptions^17–19^, pattern recognition studies on brain MRI data have only been performed in single-site studies that demonstrate substantial variability in accuracy of case-control classification between studies. A recent meta-analysis suggests that this variability may be attributable to small sample sizes, with larger studies converging at 70- 80% accuracy^15^. The latter accuracy is consistent with a recent, large-scale multi-site investigation showing reproducible brain-structural differences between individuals with schizophrenia and healthy controls^20^. These limitations in accuracy pose a significant challenge to translate psychiatric MRI tools for diagnostic and predictive applications into clinical practice. The clinical utility of such tools strongly depends on their value for everyday clinical decision making, which usually requires differential diagnosis among different disorders rather than control/case discriminations. Therefore testing diagnostic specificity is of paramount importance^21^. Bipolar disorder has particularly high differential diagnostic relevance for schizophrenia and previous studies have provided promising evidence that structural differences in schizophrenia show specificity against this disorder^22–24^. Furthermore, symptoms of attention-deficit/ hyperactivity disorder (ADHD) are among the frequent precursors of schizophrenia^25–31^ during adolescence, but have less differential diagnostic relevance in adult individuals. The three conditions show substantially shared genetic risk, and conjointly map to a spectrum of neuropsychiatric disorders with brain structure alterations associated with genetic and environmental risk factors^32^.

Based on these considerations, the collaborative FP7 project IMAging GEnetics for MENtal Disoders (IMAGEMEND) has assembled a large, multimodal database that comprises neuroimaging data on cohorts of individuals with schizophrenia and bipolar disorder, adolescent as well as adult individuals with ADHD, and healthy controls^33^. The primary focus of the project is the identification of multivariate biological signatures that can aid diagnosis of these disorders. Using this resource, we analyzed structural MRI data from 2668 individuals in the present study.

Our primary aims were 1) to identify brain structural patterns that can reproducibly differentiate individuals with schizophrenia from controls, 2) explore their diagnostic specificity with regard to other disorders and 3) to identify the underlying brain structures driving successful classification. The availability of matched case-control data from several sites allowed application of a leave-site-out procedure, meaning that data from all but one site were iteratively used for algorithm training and the remaining data used for testing. This was aimed at the identification of differences robust against between-site variability. In order to make use of the complementary information provided by the different measures, we included both 1) FreeSurfer-based measures of cortical morphometry (cortical thickness, surface area and volume) and global and subcortical volumetry as provided by Freesurfer^34^, and 2) voxel-based morphometry (VBM) as provided by Statistical Parametric Mapping (SPM)^35^. We also compared two machine learning strategies: (I) random forest machine learning, which captures non-linear and multiplicative effects of predictors and yields an efficient ranking of important predictors, and (II) support vector machines (SVM), the most commonly and successfully applied linear tool in machine learning studies on brain structure^36^.

## Materials and methods

### Cohorts

This study comprised eight cohorts with a total of 2668 participants (consisting of patients with schizophrenia (n = 375, cases in cohorts I-IV), bipolar disorder (n = 222, part of cohort VIII), ADHD (n = 342, cases in cohorts V and VI), as well as healthy control subjects (n = 1729, cohorts I to VIII; n = 368 of these in cohorts I-IV) demographic details are shown in Supplementary Table 1; recruitment details are shown in Supplementary Table 2). All participants gave written, informed consent and the study received approval from the local ethics committees of the participating institutions.

### Data pre-processing

Pre-processing of all T1-weighted images was performed centrally at the same site (University of Oslo, Norway) using FreeSurfer 5.3 (http://surfer.nmr.mgh.harvard.edu)^34^. All datasets underwent visual assessment and minor manual intervention to correct for segmentation errors wherever necessary. Data with significant low quality due to, e.g., motion artifacts and image distortions were excluded. Cortical parcellation was performed using the Desikan–Killiany atlas^37,38^, and subcortical segmentation was performed based on a probabilistic atlas^39^. The mean thickness, sum surface area, and volume for each cortical region-of-interest (ROI), as well as the volume of subcortical structures were computed, resulting in a set of 152 FreeSurfer features (Supplementary Table 4).

An important question of the present study was whether signatures that combined the effects of multiple brain structures could be represented through regionally non-specific, ‘global grey-matter features’. For this, we manually selected 20 of such ‘global features’ and these are detailed in Supplementary Table 11. Additionally, the per-subject median of all ventricle features was used as readout for global ventricle size. Furthermore, for VBM- and FreeSurfer-based analyses we determined separately the per-subject median across all features, resulting in a ‘median feature’, resulting in a set of 22 ‘global features’ in total. To avoid feature redundancy, bilateral features were removed if both uni-lateral features were available.

The dataset was also processed each using VBM^35^ as implemented in the CAT12 toolbox (http://dbm.neuro.uni-jena.de/cat/), SPM12 (http://www.fil.ion.ucl.ac.uk/spm/software/spm12/) and MATLAB 2014a (Mathworks, Sherborn, MA, USA) to derive the grey matter (GM) maps. As input, we used the *nu.mgz* volume, an intensity-normalized volume adjusted for the non-uniformity in the original T1-images, obtained from the FreeSurfer pre-processing pipeline (https://surfer.nmr.mgh.harvard.edu/fswiki/ReconAllOutputFiles). Briefly, this volume was tissue-segmented into GM, white matter (WM) and cerebrospinal fluid maps. The modulated GM maps were subsequently registered to the Dartel template, which is based on 550 healthy subjects from the IXI database (http://brain-development.org/ixi-dataset/), using affine registration followed by the Dartel non-rigid registration algorithm^40^. The mean GM density was then computed for each region-of-interest as defined in the Automated Anatomical Labeling (AAL) atlas^41^, resulting in a set of 122 VBM features (Supplementary Table 3).

### Matching, covariate adjustment and normalization

**Fig. 1:**
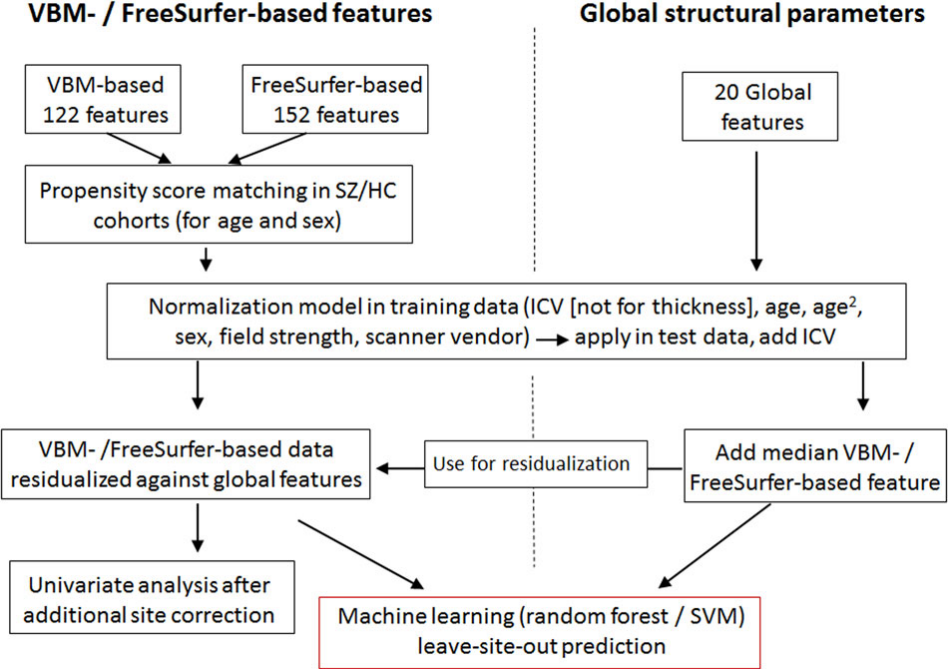
Overview of analysis procedure. Subjects were first propensity score matched and VBM- / FreeSurfer-based features were then normalized against potential confounders. Normalization models were built in training data only and these models were subsequently applied to adjust the test data. The same normalization strategy was applied for global structural parameters, which were subsequently used to remove the global structural signal from VBM- / FreeSurfer-based features. The resulting data was used for leave-site-out cross-validation analyses. For univariate analyses, as well as for machine learning analyses performed on the entire dataset, data were additionally corrected for a site factor, to account for the impact of site differences (see methods)

An overview of the pre-processing and machine learning pipeline is shown in Fig. 1. Cohorts I to IV were used for subsequent training of machine learning algorithms. In cohorts II to IV, propensity score matching (using the R library *MatchIt*^42^) was used to create schizophrenia-control datasets, 1:1 matched on age and sex. Matching was performed separately for each cohort. No matching was performed in cohort I, since it comprised fewer controls than patients and showed no significant case-control differences regarding age and sex. Controls not selected during the matching process were retained for validation of algorithms (cohort VIII).

Covariate adjustment was performed in two steps. The first step was aimed at removing the effects of covariates relevant within a given dataset. For this, linear regression was used to construct normalization models in the matched case-control data (Supplementary Figure 1). Each feature was regressed against age, age^2^, sex, and total intracranial volume (ICV, derived from FreeSurfer; this covariate was not included for thickness features derived from FreeSurfer processing). Normalization models were built separately for the cohorts used for training (i.e. during the leave-site-out procedure described below as well as for prediction of the schizophrenia classifier into the validation cohorts), and the resulting coefficients were averaged to obtain a final model per brain feature. These models were then applied to residualize the features in the training as well as the test data. Subsequently, ICV was added as a feature to the residualized training and test data. In the second covariate adjustment step, the effects of between-dataset variables (field strength and scanner vendor) were removed. Using data from the previous step as input, linear models were built to residualize all training data and adjust the test data accordingly. During the leave-site-out testing procedures, as well as for testing classifiers in validation data, the test data were not used to generate normalization models and remained independent. The objective of this two-step procedure was to appropriately account for the effect of potential confounders, without using site-information as additional covariate. This is essential for potential clinical application of a diagnostic tool, when subjects from sites are tested that are not part of the training data. In this case, adjustment against a site-covariate cannot be performed. In a secondary analysis, we set the means of each feature in a given test dataset artificially to 0 (for training data this is already fulfilled due to the residualization procedure). With this we tested whether not using test data for building of normalization models impacted on classification performance.

For the machine learning analyses performed on the entire, matched dataset (i.e. for out-of-bag performance evaluation, where accuracy estimates were obtained from observations not selected during the repeated bootstrapping part of the random forest classification procedure, see below), we excluded the impact of a site factor through residualization using linear models, in addition to the covariate adjustment described above. For this residualization, site and scanner vendor were both included as covariates. Such corrected data was also used for the univariate analyses (see below). For principal components analysis, which was applied to explore the global similarity between VBM- and FreeSurfer-based features, data were additionally normalized against diagnosis and subsequently standardized.

### Univariate analysis

Univariate analyses were performed to assess the extent of change in individual brain-structural measures prior to and following adjustment for global structural parameters. Univariate analysis was performed on data residualized as described above, to increase comparability against the features’ importance determined by machine learning. Case-control differences were evaluated using Student’s t-tests and *P*-values were adjusted for the False Discovery Rate (FDR) according to the method of Benjamini and Hochberg^43^. The adjustment was performed separately for VBM- and FreeSurfer-based features.

For the univariate analysis of the features following removal of the global structural signal, we first corrected the global structural features using the same steps described above. These corrected global structural features were then used to adjust the VBM- and FreeSurfer-based features, and the resulting residuals were used for the univariate analysis.

### Machine learning – cross-validation and accuracy estimation

Several different procedures were employed to train and test machine learning algorithms: a) ‘within-site’ classification, where algorithms were trained and tested separately in each given cohort (using cohorts I-IV for schizophrenia-control classification, cohort VIII (selecting University of Oslo data only) for bipolar disorder-control classification, and cohorts V and VI for ADHD-control classification). b) ‘Leave-site-out’ classification in cohorts I-IV. c) Prediction of a schizophrenia-control classifier in independent test data (the classifier was trained in cohorts I-IV and tested in cohorts V-VIII).

For procedures a) and b), performance of machine learning algorithms was assessed by comparing the predicted class membership against the real class-membership. For ‘within-site’ classification, this was performed using bootstrapping.

The Receiver Operating Characteristic Area Under Curve (AUC) was determined to quantify accuracy (using the R library *pROC*^44^). For leave-site-out classification, we additionally determined the mean of sensitivity and specificity to explore whether predicted class probabilities were shifted across cohorts.

For procedure c), accuracy was determined as the specificity, i.e. the percentage of subjects correctly classified as being not affected by schizophrenia.

### Machine learning – random forests

Random forest is a machine learning tool suitable for classification and regression^45^. It combines the output of a large number of individual classification/regression trees, each of which are built on randomly selected subsets of observations and predictors. The random forest can naturally incorporate interactions between predictors, allows efficient ranking of predictor importance and has been shown to be one of the most accurate classification tools on a large variety of data sets^36^.

Random forest machine learning (using the R package *randomForest*^46^) was performed in a site-stratified manner using 5000 trees and the default value for the *mtry* parameter (no tuning of random forest parameters was performed). The number of trees was chosen based on the observation that larger tree numbers do not significantly improve performance^47^. Site-stratification was performed such that for building each tree, an equal number of subjects (equal to the sample size of the smallest training cohort) were randomly drawn without replacement from the data of each site. We determined the importance of the features for prediction during this procedure using the Gini index, a measure of how much a given feature impacts the correct class separation, when used for a split during the tree-building process^48^. Selection of the most important predictors was performed using the R package *varSelRF*^49^, also using 5000 trees, and default settings otherwise. During this procedure, the least important variables are successively removed from the model. The optimal number of variables is chosen for the solution where the out-of-bag error is equal to the lowest observed error rate, plus one standard deviation. This leads to a solution with close to optimal error rate but with a lower number of predictors, a scenario generally thought to be beneficial for the generalizability of the classifier. The Gini-index-derived variable importance measure was also used to assess the similarity of features selected by within-site classification. For this, we determined the median Pearson correlation of the variable importance measures across cohorts.

To explore the diagnostic specificity of important variables, we first selected the top *m* (with m being determined via random forest variable selection; *m* = 14 for VBM-based and m = 11 for FreeSurfer-based features, respectively) variables from the schizophrenia-control comparison. We then determined the Wilcoxon rank sum statistic comparing the importance of these variables against the remaining variables in bipolar disorder, adolescent as well as adult ADHD. To test significance, a 5,000-fold permutation of diagnostic labels was performed. During each repetition, variable importance was re-calculated for the three non-schizophrenia case-control comparisons and the determination of rank sum statistics was repeated. Empirical *P*-values were then calculated as the frequency of permutation rank sum statistic at least as high as those determined from non-permuted data.

Random forest regression was used to determine the amount of variance that could be predicted in individual VBM- and FreeSurfer-based features using the global structural parameters. The explained variance was determined from out-of-bag predictions. For this analysis, the same covariate-adjusted data were used as for the univariate analysis (see above). Accordingly, the global structural parameters were also additionally residualized against a site factor.

### Machine learning – Support Vector Machines

A support vector machine is a classification tool that aims to identify a decision boundary with maximal margin between the boundary and observations from a given class^50^. The boundary is defined based on the most proximal observations, making classification insensitive to data variations or outliers, resulting in frequently superior generalization performance^36^. Linear SVM is relatively robust to overfitting and was, in the present study (using the R package *e1071*^51^), tuned using 10-fold cross-validation to optimize the cost parameter (choosing among values from the log sequence between 10^−5^ and 10^5^). Parameter optimization was performed in training data only.

### Exploring the impact of global structural parameters on classification

To explore the effect of the 22 global structural features on classification, these features were adjusted for confounding variables using the same procedure applied for VBM- and FreeSurfer-based features (i.e. residualization against age, age^2^, sex, gender, ICV, field strength, and scanner vendor). VBM- and FreeSurfer-based features were subsequently residualized against the covariate-adjusted global features using additive linear models. To explore the impact of this residualization procedure *per se*, it was repeated 1000 times with row order-permuted global features. Similarly, to explore the significance of the accuracy obtained after residualization, the procedure was repeated 1000 times with permuted diagnostic labels. Finally, to explore the classification accuracy obtained from global-features only, we applied random forest machine learning (as described above) using the covariate-adjusted global features.

## Results

Brain structural neuroimaging data from a total of 2668 subjects were analyzed. Sample details are presented in Supplementary Tables 1 and 2. The data were processed to extract either 122 VBM-based or 152 FreeSurfer-based morphometry features (Fig. 1, Supplementary Tables 3 and 4, ICV was added as a predictor to each feature set). Machine learning was used to identify structural patterns that could be used to differentiate individuals with schizophrenia from controls and to establish the diagnostic specificity against bipolar disorder and ADHD.

### Case-control differences, schizophrenia classification and diagnostic specificity *Univariate case-control differences*

the univariate analysis of matched cases and controls from cohorts I to IV demonstrated significant alterations in VBM-based features of individuals with schizophrenia (Supplementary Tables 3 and 4). A total of 110 of the 123 features showed significant alteration at FDR < 0.05. Similarly, for FreeSurfer-based features, 105 of the 153 features were significant at this threshold.

### *Machine-learning classification*

Using random forest machine learning, we first performed a within-site classification of participants with schizophrenia and controls and found AUC values obtained from out-of-bag predictions ranging from 0.58 to 0.82 for VBM-based and from 0.58 to 0.80 for FreeSurfer-based features, respectively (Supplementary Table 5). Permutation analysis showed that accuracy estimates were significant for three of the four cohorts (Supplementary Table 5). When all case-control cohorts were combined into a single dataset, the AUC obtained from out-of-bag predictions was 0.73 (*P* < 0.001) for VBM-based and 0.72 (*P* < 0.001) for FreeSurfer-based morphometry, respectively. When VBM- and FreeSurfer-based features were combined into a single dataset, the resulting AUC was 0.74 (*P* < 0.001). We further found that features were more consistently selected as important predictors for VBM data (median correlation of variable importance measures across the four cohorts of 0.11) compared to FreeSurfer data (mean correlation -0.02).

### *Leave-site-out classification*

**Fig. 2:**
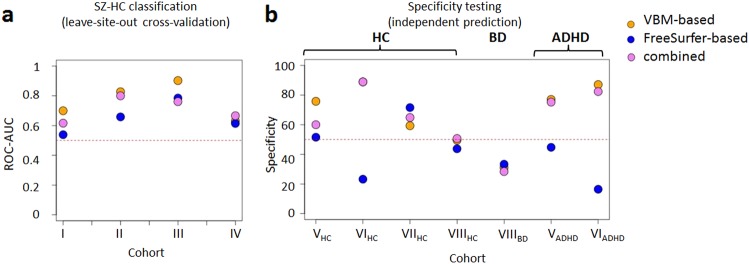
Accuracy of schizophrenia classifier using VBM- and FreeSurfer-based morphometry features. **a**) Leave-site-out cross-validation performance measured as the ROC-AUC. **b** Specificity of schizophrenia-control classifier (trained on all SZ-HC cohorts) for prediction in independent cohorts. The red horizontal line demonstrates 50% ROC-AUC or specificity, respectively. The classification was based on random forest machine learning. SZ: schizophrenia; BD: bipolar disorder; ADHD: attention-deficit/ hyperactivity disorder; HC: healthy controls

We tested the classification accuracy when all but one of the case-control datasets were used for training. This leave-site-out cross-validation yielded median AUC estimates of 0.76 (range 0.63 to 0.90) and 0.64 (range 0.54 to 0.78) for VBM- and FreeSurfer-based morphometry features, respectively. The median AUC for the combined feature set was 0.71 (range 0.62 to 0.80) (Fig. 2a). For VBM-based data, the observed accuracy corresponded to a sensitivity-specificity mean with a median of 0.70 across cohorts I-IV. We observed that sensitivity and specificity varied substantially across cohorts (Supplementary Table 6). In FreeSurfer-based data, this was even more pronounced with a corresponding estimate of 0.52, showing that the optimal cut-off for classification differed across cohorts (Supplementary Figure 2). This was likely due to shifts of structural volume means across cohorts. The normalization models aim to set structure mean values in the test data to zero, but this is not guaranteed as test data were not used for building the normalization models. Setting test data means to zero (a strategy commonly employed in machine learning) resolved the sensitivity-specificity imbalance (sensitivity-specificity mean with a median of 0.76, 0.71 and 0.71 for VBM-, FreeSurfer and combined data, respectively. AUC values were 0.79, 0.75 and 0.78, respectively; see Supplementary Table 7).

### *Specificity testing in independent test cohorts*

For VBM-based features, the application of an algorithm trained on all four training cohorts resulted in accuracies ranging from 50% to 89% (median 68%) in four independent cohorts of healthy controls (Fig. 2b, Supplementary Table 8). The algorithm showed limited specificity against bipolar disorder as 69% of the 222 individuals were assigned to the schizophrenia class. To explore potential associations between prediction accuracy and the presence of psychotic features among individuals with bipolar disorder, we identified subsets of individuals with severe psychosis (n = 28) and individuals without psychotic features (n = 48). However, we found no evidence that accuracy significantly differed between these clinical groups (*P* = 0.63).

In contrast, when applying the algorithm to adult (n = 85) and adolescent (n = 257) subjects with ADHD, schizophrenia classification showed similar accuracy (87% and 77% correctly classified as not belonging to the schizophrenia class) as for healthy control subjects. Notably, classification based on FreeSurfer-based morphometry features showed substantially poorer accuracy in most independent validation cohorts (Fig. 2b, Supplementary Table 8). As for leave-site-out classification, this was due to mean shifts of covariate-adjusted data that affected FreeSurfer-based morphometry features important for schizophrenia classification and is exemplified for amygdala volumes in Supplementary Figure 3.

### *Comparison between classifier types*

To explore whether prediction results were influenced by the choice of the algorithm, we replaced the site-stratified random forest with a non-site-stratified, linear SVM. This showed that across all conducted tests, SVM outperformed random forest classification by a small margin (Supplementary Table 6, Supplementary Figure 4). Notably, linear SVM application also showed an improved specificity of the schizophrenia classification against bipolar disorder (specificity between 48 and 55%, Supplementary Table 6, Supplementary Figure 4).

### *Case-control classification of differential diagnoses*

VBM-based data showed limited utility for a meaningful differentiation of bipolar disorder (AUC of 0.63, derived from random forest out-of-bag prediction), adult (AUC = 0.58), or adolescent (AUC = 0.62) ADHD from healthy controls within the respective, propensity score-matched cohorts. On the same cohorts, similar performance estimates (AUC of 0.66, 0.56, and 0.63 respectively) were obtained for FreeSurfer-based features.

### Exploration of features important for classification

**Fig. 3:**
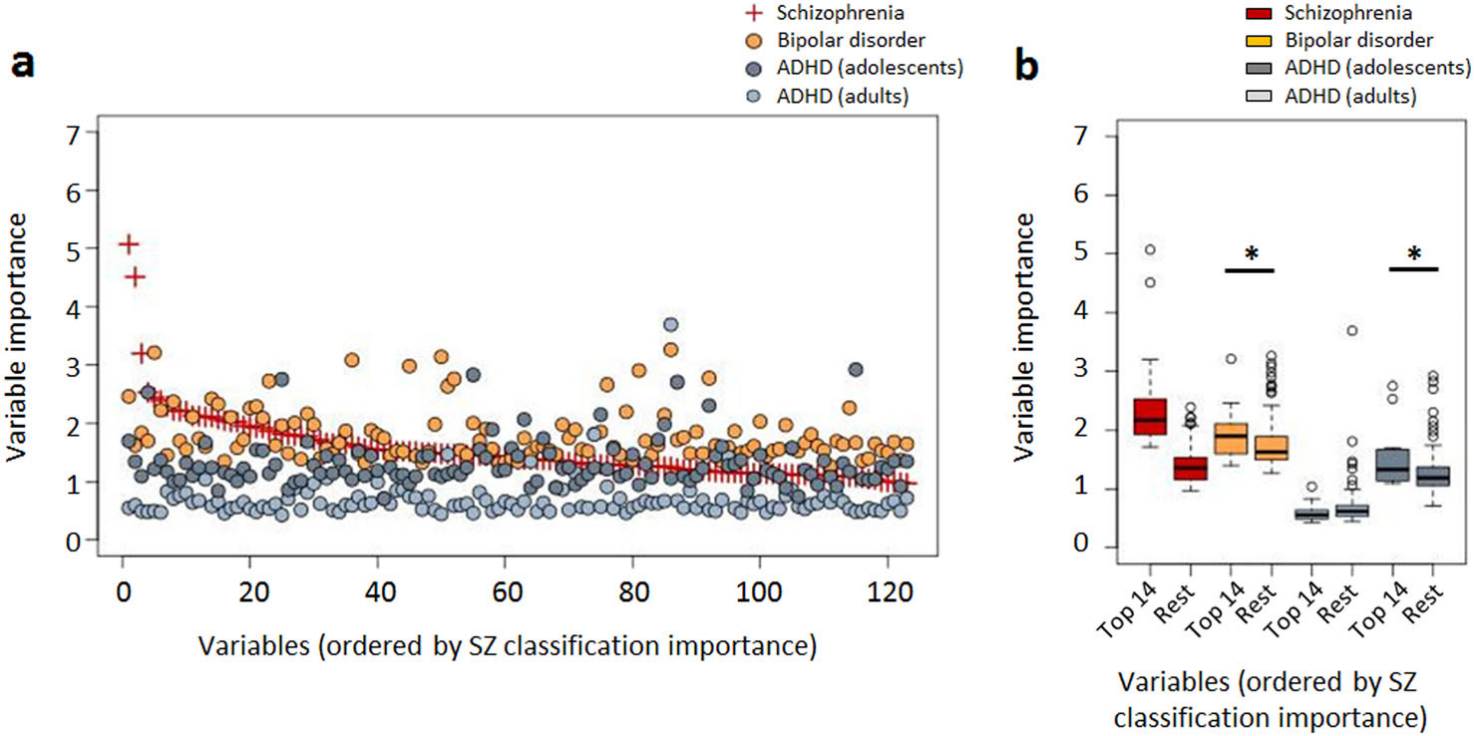
VBM-based variable importance for classification. **a** Random-forest variable importance for the schizophrenia vs. control (red, used to order the x-axis), the bipolar disorder vs control and the ADHD vs control comparisons. **b** Boxplot of random-forest variable importance measures, comparing the 14 most important schizophrenia predictors against the remaining predictors in bipolar disorder and ADHD. The asterisk indicates significance determined from permutation testing. Since variable importance was determined from the schizophrenia-control comparison, no significance estimate is shown for the corresponding boxplot

The random forest variable importance derived from the site-stratified classifiers based on all case-control cohorts was used to identify the features most relevant for classification. The ranked variable importance measures derived from VBM-based morphometry data are shown in Fig. 3a (and Supplementary Table 9). Using random forest feature selection, we found 14 VBM-based features (11 for FreeSurfer-based data) to be of particular importance for classification, i.e. the respectively smallest feature sets leading to the minimum error rate plus one standard deviation (see methods). Figure 3a further displays the importance of VBM-based features for classification of bipolar disorder (propensity score-matched patients and controls from University of Oslo bipolar disorder and control data part of cohort VIII, n = 444) and ADHD (propensity score-matched patients and controls from cohorts V (adolescent subjects), n = 322, and VI (adult subjects), n = 170). The top 14 features for schizophrenia-control classification had also significantly higher importance for bipolar disorder-control as well as the adolescent subjects with ADHD vs. controls classification (*P* = 0.011 and *P* = 0.008, respectively; permutation test, Fig. 3b), compared to the remaining features. In contrast, these features were of no significant importance for the adult ADHD-control classification (*P* = 0.857, Fig. 3b). Supplementary Figure 5 displays the variable importance measures derived from FreeSurfer-based morphometry data (Supplementary Table 10), showing a similar pattern for schizophrenia markers and those for bipolar disorder (*P* = 0.003) as well as adult (*P* = 0.196) ADHD compared to VBM-based analysis. Notably, for FreeSurfer-based morphometry data, no overlap with adolescent ADHD markers was found (*P* = 0.350).

### Relation between VBM-based and FreeSurfer-based predictors

**Fig. 4:**
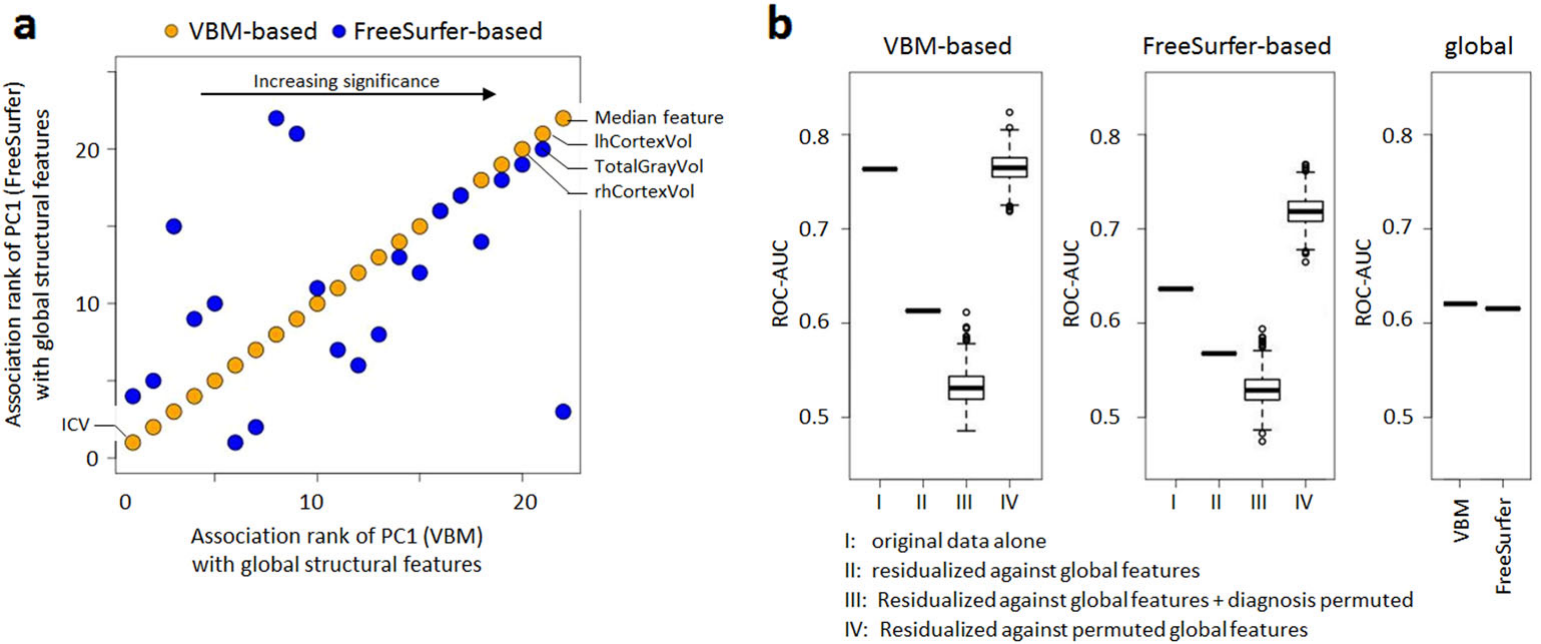
Effect of global structural covariates on classification. **a** Comparison of associations between global structural features and the first principal components determined from the 14 selected VBM-based (orange; used to order the x-axis) and the 11 selected FreeSurfer-based (blue) features (see also Supplementary Table 1,0). **b** Effect of residualization against global structural features on classification performance and classification performance obtained from global features only. Notably, AUC values obtained from analyses with permuted diagnoses showed mean values > 0.5, which was due to chance associations in the comparatively small datasets. Furthermore, surface based features showed an increase in performance after residualization against permuted global features. This suggests features with poor cross-site reproducibility were coincidentally prioritized for classification in the original data and this was remedied in the residualized data. The two sets of global features were identical except for the addition of either a median VBM- or FreeSurfer-based feature

Between the top-14 VBM-based and the top-11 FreeSurfer-based predictors for the schizophrenia-control classification, we found significant pairwise correlations (median Pearson’s correlation coefficient of 0.16, using subjects from cohorts I to IV, after additional residualization against diagnosis). Accordingly, in this confounder-corrected dataset, the first principal components (PCs) of the top features (explaining 42% and 38% of variance in FreeSurfer-based and VBM-based features, respectively), were strongly correlated (ρ = 0.43, *P* = 5.4·10^−34^). This raised the question whether the numerous, individually weak structural predictors were related to a common global measure of brain structure. To explore this, we tested associations between the principal components and 22 global measures of brain structure and found highly significant correlations with the large majority of these measures (Fig. 4a, Supplementary Table 11). This effect was not due to residual confounding of any PC by total intracranial volume, age, age^2^, sex, scanner vendor, field strength or recruitment site (all uncorrected *P* > 0.12).

### Effect of global structural parameters on classification and univariate differences

We then explored, whether these global measures explained part of the multivariate signal that allowed case-control differentiation between patients and controls. Figure 4b shows that residualization of VBM- and FreeSurfer-based features against the 22 global measures led to a decrease in classification performance (measured as the leave-site-out AUC determined on cohorts I to IV) from 0.76 to 0.61 (VBM-based) and from 0.64 to 0.57 (FreeSurfer-based), respectively. These AUC values were close to (VBM-based) or within (FreeSurfer-based) the range of those obtained after randomly permuting diagnostic grouping (Fig. 4b). Accuracy did not decrease substantially, when residualization was performed with permuted global covariates, showing that residualization against large covariate numbers did not per se have a substantial impact (Fig. 4b). Classification using covariate-corrected global features alone led to a leave-site-out AUC of 0.62, regardless of whether the median VBM- or the median FreeSurfer-based feature was included (Fig. 4b). This raises the question why global structural features were strong co-variates of case-control associations, but relatively poor predictors of diagnostic status when used alone. This effect was likely due to site-to-site variability of the global structural features, since random forest learning applied on the entire dataset yielded out-of-bag AUC values of 0.71 for both global structural parameter sets. These values were comparable to the out-of-bag estimates derived from similarly corrected VBM- (AUC = 0.73) or FreeSurfer-based (AUC = 0.72) features. This further supports the extent of signal shared between global features and individual brain structures.

Notably, the residualization against global features also led to substantial decrease in univariate significance (Supplementary Table 3). For VBM-based features, after residualization, FDR-corrected significance was only observed for a bilateral increase in the pallidum (left: *P*_*FDR*_ = 2.5·10^−5^; right: *P*_*FDR*_ = 1.5·10^−4^) and a decrease in the right hippocampus (*P*_*FDR*_ = 0.026). For FreeSurfer-based features, after residualization against global parameters, no significance was observed.

### Prediction of individual structural features through global structural parameters

We explored whether individual brain structural features could be accurately predicted based on global structural parameters. Based on random forest regression, the global features explained a mean of 29% ± 13 (range 2.5% – 61.2%) of variance in VBM-based features and a mean of 29% ± 15 (range 0.0% – 64.8%) of variance in FreeSurfer-based features, respectively (Supplementary Tables 3 and 4). In VBM-based data, the variance explained by global features was further correlated with the mean size of the respective structure (ρ_VBM_ = 0.33; *P*_*VBM*_ = 0.0002; ρ_surface_ = −0.06; *P*_surface_ = 0.44; Spearman correlation, to prevent overdue influence of larger structures).

## Discussion

The primary findings of this multi-site investigation were 1) the presence of reproducible brain-structural patterns that could differentiate individuals with schizophrenia from healthy controls, 2) the specificity of the patterns when applied on data from individuals with ADHD, and the lack thereof in bipolar disorder, 3) the significant overlap of markers important for classification of schizophrenia, bipolar disorder and adolescent ADHD and 4) the finding that brain-structural changes were strongly associated with global structural parameters.

Based on brain-structural patterns, individuals with schizophrenia could be reproducibly differentiated from healthy controls, with a median AUC of up to 0.76. Performance estimates were derived from unbiased leave-site-out cross-validation and no test set data were used to determine parameters of covariate adjustment or machine learning models. Therefore, the obtained estimates are likely to reflect the performance of the algorithms, when tested in independent data. We observed that when test data were not used during generation of normalization models, sensitivity and specificity fluctuated substantially, which could be resolved by scaling of the test data. This, however, would require at least some data from a given test site to be available prior to testing algorithms in data from that site^20^. It should also be noted that biological heterogeneity resulting from the current diagnostic system limits the accuracy biological predictions can achieve, when aiming to reproduce clinical classifications, constituting a general caveat for the field.

The brain-structural patterns associated with schizophrenia showed significant lack of specificity against bipolar disorder, consistent with the substantial genetic and clinical overlap of the two disorders^30,31,52^. Notably, the signatures were specific against adolescent and adult ADHD. Subjects with ADHD, did not, however, show brain-structural alterations that could be used for accurate classification, nor did those with bipolar disorder. Despite this, the VBM-based feature sets most useful for classification of adolescent ADHD and schizophrenia showed significant overlap. Given the high specificity of the schizophrenia classifier against adolescent ADHD, this supports divergent profiles in the same feature set. A particular strength of the present study was that conclusions regarding differential diagnostic specificity against bipolar disorder were not confounded by site variability. Considering the observed specificity fluctuations during leave-site-out testing, it should, however, be noted that the preferential classification of subjects with ADHD as controls could have been influenced by between-site effects. Similarly, non-specificity of the schizophrenia classifier against bipolar disorder was determined in one cohort and requires further replication. Also, the lack of adolescent subjects in the training data may have confounded the accuracy observed in adolescent ADHD subjects.

We aimed to identify brain-structural features driving reproducible schizophrenia-control classification and to compare these between two different pre-processing strategies. We observed that these strategies led to identification of differential structural patterns but found that these alterations were, to a large extent, capturing overlapping global brain-structural alterations. Removing variation explained by measures of global structural properties also removed most of the identified multivariate signals. Notably, global structural parameters were strong confounders of VBM- and FreeSurfer-based feature associations, but were on their own relatively poor predictors of diagnosis. Our results indicate that this was, to a significant extent, due to between-site variability affecting the global signal. This effect may be due to the fact that the global signal combines multiple signals that are individually affected by site-specific effects (such as the shifts in mean measurement observed in the present study), creating an aggregate signal reflecting site idiosyncrasies. This, in turn, raises the important question to what extent global variables reflect the underlying biology vs. measurement factors (i.e. the signal to noise ratio) in structural imaging data. The observed case-control classification performance is consistent with previous large-scale analyses^15,20^, thus it is unlikely that measurement uncertainty specific to the present study accounts for the global effects detected. Furthermore, GM differences have been observed in numerous studies investigating first-episode schizophrenia patients, suggesting that these effects are not primarily related to the specific clinical characteristics of the samples we examined [e.g^53–55^]. One possible interpretation of these results is that schizophrenia entails a combination of isometric and allometric structural changes which may vary between individuals and within patients across different stages of the illness. This explanation may account for the low effect sizes and effect heterogeneities of structural differences previously observed in schizophrenia. Another interpretation is that a shared biological component affecting global variables across multiple disorders discriminates controls from cases, but does not differentiate patients with different diagnoses. Accordingly, previous reports highlighted shared genetic components across multiple psychiatric disorders and personality traits^56,57^. In contrast, the present results may also be interpreted from the perspective of cross-cohort reproducibility. That is, the reduction in classifier accuracy through consideration of global structural features primarily relates to effects on reproducible alterations in GM features. Changes in individual sites, in contrast, may have persisted despite the normalization against the global signals. This interpretation raises the question whether this and previous studies had sufficient resolution, in view of the large site to site differences, to investigate reproducible regional effects. An improved imaging resolution could also allow identifying patterns of structural differences that show higher specificity between schizophrenia and bipolar disorder. A corollary of this view is the question whether, even assuming that structural imaging resolution yields sufficient signal to noise ratio to study regional effects, the correlations between regional and global variables caused by common underlying biology and by shared measurement uncertainties can be meaningfully disentangled. For example, we found that identification of univariate changes was strongly dependent on global structural alterations. Importantly, if the global signal was indeed more affected by site specific experimental effects than individual brain structures, it would be challenging for single-site investigations or univariate statistics to appropriately account for this effect, limiting the possibility to reproduce findings across studies.

In this context, a limitation of the present study is the lacking incorporation of other data modalities, such as demographic, clinical or psycho-behavioral features, which could potentially have informed on the presence of patient subgroups or illness-dimensions in relation to brain-structural alterations. Similarly, future studies should explore the effects of antipsychotic treatment on GM, which have been observed in schizophrenia (i.e. ref. ^9^) and are supported by data from animal models^58,59^, but which have also been found in antipsychotic-native subjects^9^. An acerbation of disorder-intrinsic structural changes by medication may be a possible explanation why removal of the global signal almost completely removed structural differences. While this study explores the impact of different pre-processing strategies on machine learning analysis of brain-structural differences, it does not offer a comprehensive analysis of the broad spectrum of preprocessing methods currently available. The sensitivity of machine learning to the choice of preprocessing may contribute to the variability of such analyses as reported in previous studies. Another limitation of the present study is the fact that it involved already diagnosed patients. One of the most significant aspects of clinical utility will be the ability to accurately predict the transition from early signs to full-blown illness, such that appropriate treatment can be started earlier.

Finally, an interesting finding was that linear SVM application showed marginally better classification performance compared to RF machine learning. This suggests that classification did not profit from RF’s ability to model complex interactions. Interestingly, schizophrenia classification using linear SVM also showed an improved specificity against bipolar disorder, which requires further validation in independent cohorts.

In conclusion, this study identified reproducible GM patterns that index a multivariate, global alteration of brain structure in schizophrenia and bipolar disorder, but are different from those seen in ADHD. These results may reflect the biological heterogeneity of schizophrenia and are consistent with previous observations of shared genetic determinants between these disorders. The results further demonstrate the need for appropriately accounting for the global signal during analysis of individual brain structures. They underline the importance of biologically dissecting these illnesses as a basis to redefine diagnostic boundaries using biological parameters. These efforts may benefit from integrative analyses of other relevant data modalities, including genetic risk measures or functional neuroimaging, which may yield more accurate and specific classifiers that have clinical utility. Also, substantial differences in the ability to derive reproducible brain-structural signatures were found when using VBM or FreeSurfer features derived from the same individuals, highlighting the importance of preprocessing strategies for machine learning analysis of brain-structural data. Finally, the present results highlight the need for a more in-depth analysis of how individual brain structures contribute to the pathophysiology of these psychiatric disorders.

### Code availability

Code used for the analyses described in this manuscriptis available from the corresponding author upon request.

## Supplementary information

Supplementary Figures

Supplementary Tables
