# Supplementary material for: Reproducible grey matter patterns index a multivariate, global alteration of brain structure in schizophrenia and bipolar disorder
Source: Transl Psychiatry. 2019 Jan 17;9:12. doi: 10.1038/s41398-018-0225-4 (PMC6341112; doi:10.1038/s41398-018-0225-4)
Supplement: Supplementary file 1 — Supplementary Figures [file 41398_2018_225_MOESM1_ESM.docx]

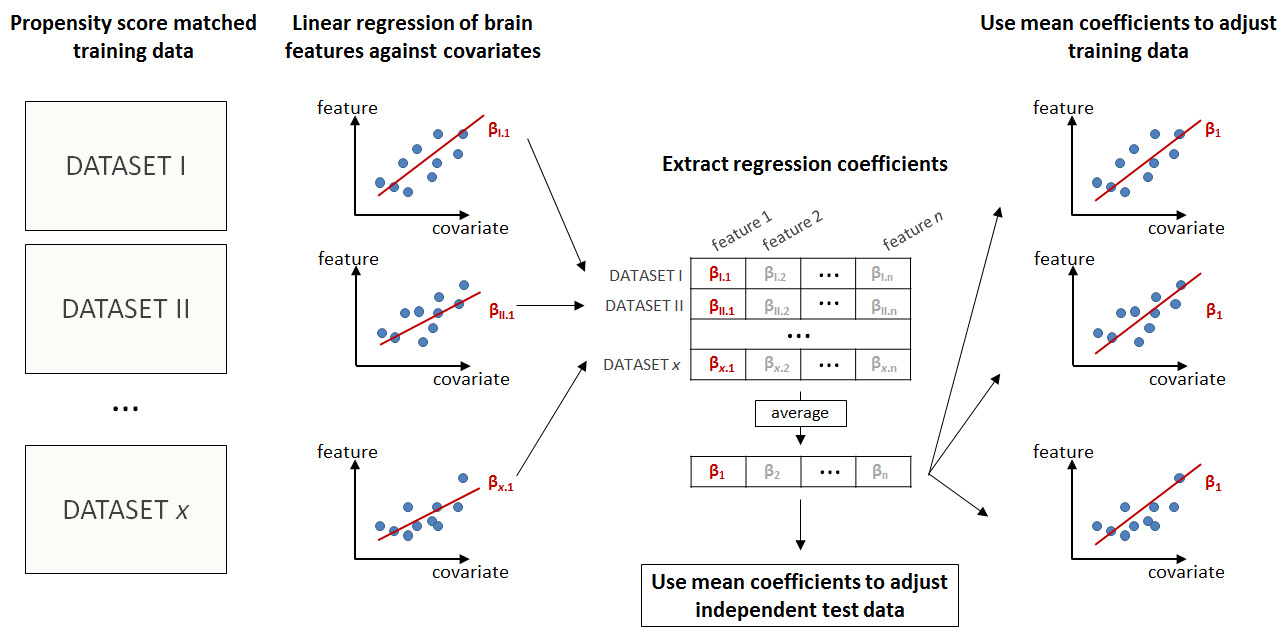


**Supplementary Figure 1. Schematic overview of covariate adjustment procedure.** Regression coefficients were calculated for each dataset separately and then averaged. The mean coefficients were used to adjust the training data as well as the independent test data.


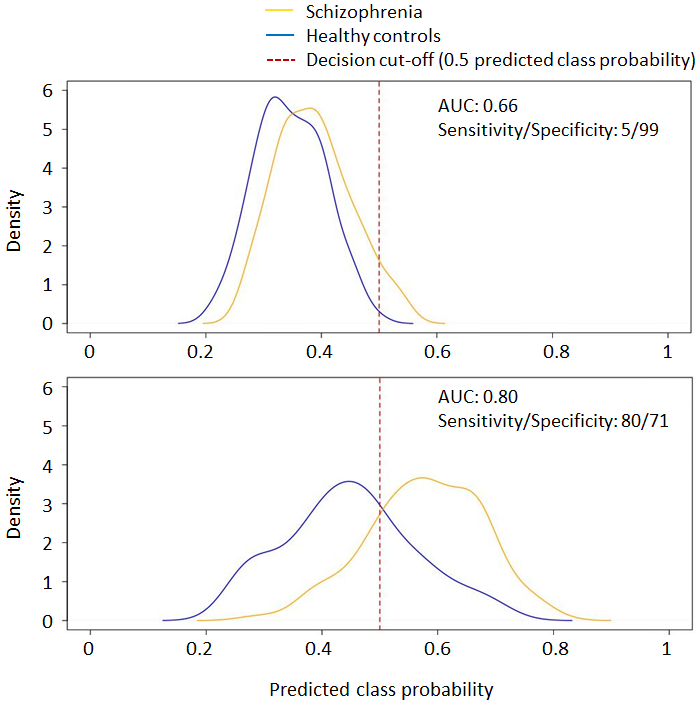


**Supplementary Figure 2. Comparison of predicted class probabilities without (top panel) and with (bottom panel) additional scaling of the test data.** This example is based on FreeSurfer-based features from cohort II, using random forest classification. Subjects with scores to the right of the red vertical line are classified as individual with schizophrenia.


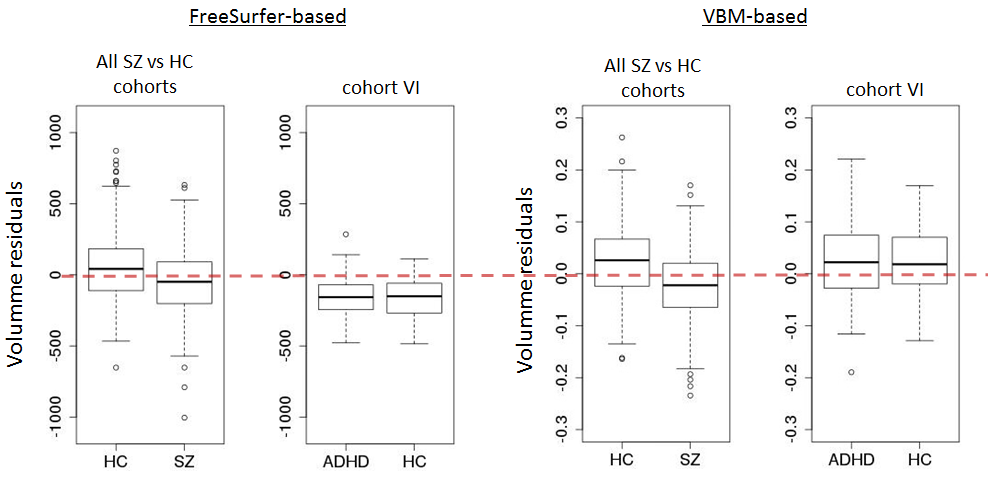


**Supplementary Figure 3. Mean shift of left amygdala volume in cohort VI between FreeSurfer-based and VBM-based preprocessing.** While both feature types show decreased volumes in patients, the mean volume is shifted to lower values for surface-based preprocessing, resulting in subjects being preferentially classified as patients.


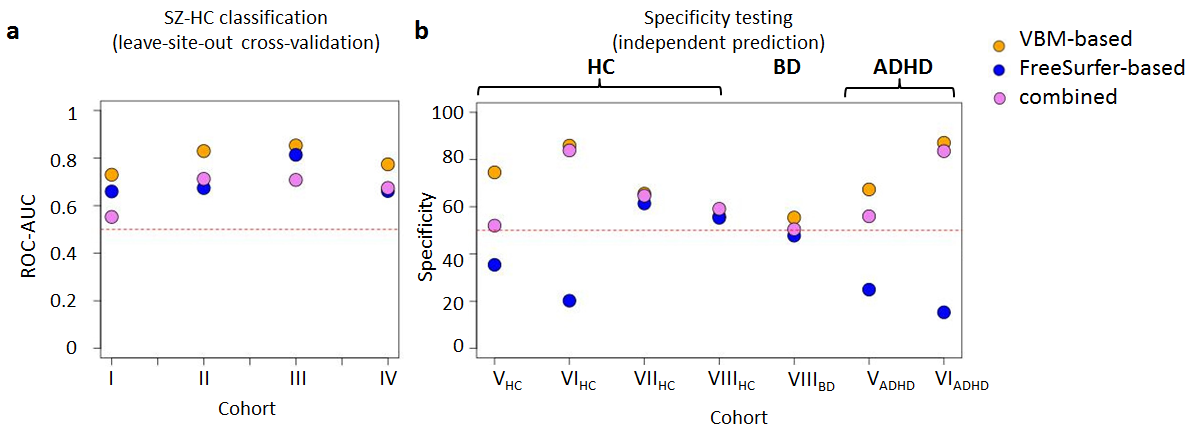


**Supplementary Figure 4. Accuracy of support vector machine classifier using VBM- and FreeSurfer-based morphometry features. a)** Leave-site out cross-validation performance measured as the ROC-AUC. **b)** Specificity of schizophrenia-control classifier (trained on all SZ-HC cohorts) for prediction in independent cohorts. The red horizontal line demonstrates 50% ROC-AUC or specificity, respectively.


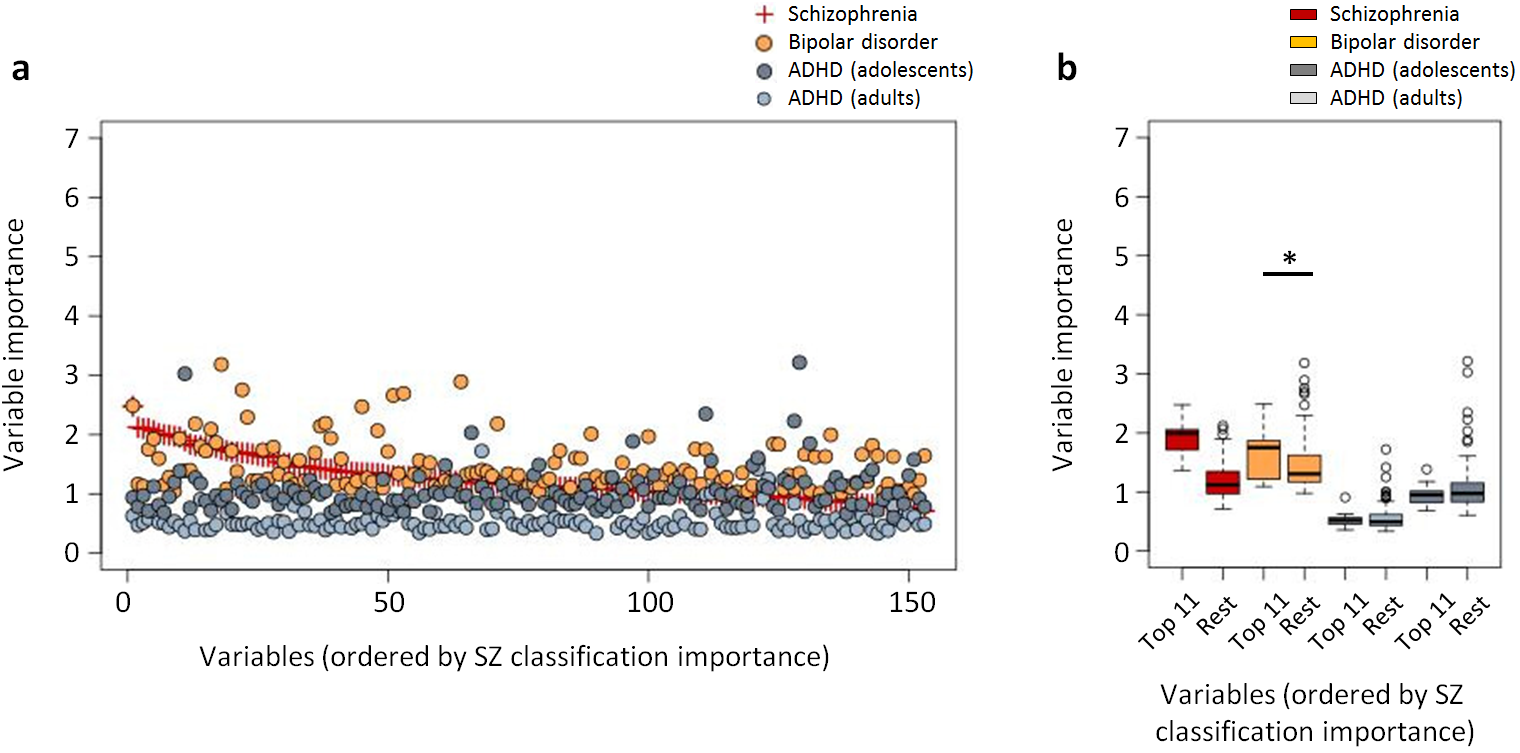


**Supplementary Figure 5. FreeSurfer-based variable importance for classification. a)** Random-forest variable importance for the schizophrenia vs. control (red, used to order the x-axis), the bipolar disorder vs control and the ADHD vs control comparisons. **b)** Boxplot of random-forest variable importance measures, comparing the 11 most important schizophrenia predictors against the remaining predictors in bipolar disorder and ADHD. The asterisk indicates significance determined from permutation testing. Since variable importance was determined from the schizophrenia-control comparison, no significance estimate is shown for the corresponding boxplot.
