# Supplementary material for: Reproducible grey matter patterns index a multivariate, global alteration of brain structure in schizophrenia and bipolar disorder
Source: Transl Psychiatry. 2019 Jan 17;9:12. doi: 10.1038/s41398-018-0225-4 (PMC6341112; doi:10.1038/s41398-018-0225-4)
Supplement: Supplementary file 2 — Supplementary Tables [file 41398_2018_225_MOESM2_ESM.docx]

**Supplementary Table 1: Discovery and validation cohorts used in the present study**

| **Cohort / site** | **Scanner/field strength** | **Dx** | **n**  **patients** | **n**  **HC** | **Age**  **patients** | **Age HC** | **Sex patients** | **Sex HC** |
| --- | --- | --- | --- | --- | --- | --- | --- | --- |
| **Discovery** |  |  |  |  |  |  |  |  |
| I / CIMH | Siemens / 3T | SCZ, HC | 51 | 44 | 31.0 ± 8.6 | 30.4 ± 11.5 | 34/17 | 29/15 |
| II / HUBIN/KaSP | GE / 1.5T | SCZ, HC | 94 | 94 | 41.7 ± 7.6 | 41.8 ± 8.9 | 70/24 | 67/27 |
| III / UiO | Siemens / 3T  GE / 1.5T | SCZ, HC | 164 | 164 | 31.7 ± 8.7 | 31.6 ± 8.7 | 105/59 | 105/59 |
| IV / UNIBA | GE / 3T | SCZ, HC | 66 | 66 | 33.4 ± 8.1 | 32.9 ± 8.6 | 46/20 | 50/16 |
|  |  |  |  |  |  |  |  |  |
| **Validation** |  |  |  |  |  |  |  |  |
| V / NeuroIMAGE | Siemens / 1.5T | ADHD, HC | 257 | 161 | 17.3 ± 3.2 | 16.7 ± 3.2 | 181/76 | 68/93 |
| VI / IMPACT | Siemens / 1.5T | ADHD, HC | 85 | 99 | 32.6 ± 9.4 | 35.4 ± 11.7 | 24/61 | 25/74 |
| VII / UBA | Siemens / 3T | HC | - | 580 | - | 22.6 ± 3.4 | - | 215/365 |
| VIII / Discovery sites* |  |  |  |  |  |  |  |  |
|  | GE /1.5T (cohort II) | HC | - | 6 | - | 47.7 ± 0.6 | - | 0/6 |
|  | Siemens / 3T  GE / 1.5T (cohort III) | BD, HC | 222 | 278 | 33.9 ± 11.3 | 33.6 ± 9.0 | 92/130 | 141/137 |
|  | GE (cohort IV) | HC | - | 237 | - | 24.9 ± 6.1 | - | 89/148 |

* subjects not selected during propensity score matching

**Supplementary Table 2. Recruitment details of investigated cohorts.**

| **Cohort** | **Diagnoses** | **Details** |
| --- | --- | --- |
| I | SZ, HC | Recruitment procedures for this cohort have been described elsewhere [see ^1, 2^]. In short, subjects were recruited at the Central Institute of Mental Health, Mannheim, Germany and comprised two different sets of adult schizophrenia patients: I) first episode patients according to DSM-IV ^3^ criteria (n=16) and II) chronic schizophrenia patients (n=29) according to DSM-IV-R criteria. For the first cohort, exclusion criteria included age > 40 years, insufficient German language skills, intake of antipsychotic medication for > 4 weeks in total or within 4 weeks prior to testing, substance dependence (other than nicotine), or disorders of the CNS requiring treatment. For the second cohort, exclusion criteria included age > 60 years, insufficient German language skills, exacerbated manifestation (Positive and Negative Syndrome Scale (PANSS) score ≥ 90), current substance dependence (other than nicotine) or other disorders of the central nervous system requiring treatment, and treatment with multiple antipsychotics. Exclusion criteria for controls included presence of past psychiatric disorders [assessed using the Mini-International Neuropsychiatric Interview (M.I.N.I.) ^4^], former or present psychopharmacological treatment and abuse of illegal substances within 4 weeks prior to testing. |
| II | SZ, HC | Recruitment details for this cohort have been described previously [see ^5-7^]. Briefly, subjects were recruited as part of the Human Brain Informatics (HUBIN) project and Karolinska Schizophrenia Project (KaSP) in Stockholm, Sweden. In HUBIN, patients fulfilled DSM-IIIR or DSM-IV criteria for schizophrenia based on hospital case notes and structured clinical interviews ^8, 9^. Healthy controls were recruited from population registries or among hospital staff. Exclusion criteria for controls were presence of severe mental disorders among first-degree relatives, history of head trauma with loss of consciousness for more than 5 min, or somatic disorders affecting brain function. In KaSP, diagnosis was established based on a structured clinical interview of the DSM-IV or a consensus diagnostic procedure^7^. Exclusion criteria were neurologic or severe somatic illness, substance abuse and autism spectrum disorder. Patients with more than 1 month of treatment with antipsychotics were not included in the study. Healthy control subjects were recruited by advertisement. Medical examination was made by routine laboratory blood and urine tests, as well as physical examination. The Mini International Neuropsychiatric Interview was used to exclude previous or current psychiatric illness. Further exclusion criteria were previous or current use of illegal drugs and first-degree relatives with psychotic illness. |
| III and VIII | SZ, BD, HC | Recruitment procedures for this cohort have been described previously [see ^10^]. Briefly, participants were recruited as part of the Thematically Organized Psychosis (TOP) study in Oslo, Norway. Diagnoses were established based on the Structured Clinical Interview for DSM-IV Axis I Disorders (SCID) ^9^. Exclusion criteria included age > 65 years, inability to understand and speak Scandinavian language, history of severe head trauma and IQ ≤ 70. Exclusion criteria for healthy controls included the presence of a severe psychiatric illness in first-degree relatives. Controls were sampled from national registries from the same catchment area and age range as the patients, had no psychiatric or alcohol/substance use disorder, as well as no cannabis use for the last 3 months. |
| IV | SZ, HC | This cohort has been described in detail elsewhere [see ^11^]. In short, subjects were recruited at the University Hospital of Bari and University Hospital of Verona (Italy). Patients fulfilled schizophrenia criteria as defined in DSM-IV-TR. Exclusion criteria for cases and controls included a history of drug or alcohol abuse in the past year, non-psychiatric clinically relevant conditions, history of neurological diseases and head trauma with loss of consciousness. Absence of psychiatric illness in HC was established using the Structured Clinical Interview for DSM-IV (SCID). |
| V | ADHD, HC | This cohort comprised subjects from the NeuroIMAGE project ([www.neuroimage.nl](http://www.neuroimage.nl)), the Dutch follow up of the International Multicenter ADHD Genetics (IMAGE) study ^12^. Recruitment details have been described previously [see ^13, 14^]. Briefly, ADHD subjects were required to have six or more hyperactive/impulsive and/or inattentive symptoms according to DSM-IV criteria; controls had to have less than two symptoms overall, based on a structured psychiatric interview (K-SADS) and Conners questionnaires. The control cohort comprised unaffected relatives as well as unrelated healthy subjects, which were treated as one control group in the present study. Exclusion criteria included the presence of a “subthreshold ADHD diagnosis”, since these subjects could not unambiguously attributed to any diagnostic group. |
| VI | ADHD, HC | Participants had previously been recruited as part of the International Multicentre persistent ADHD CollaboraTion (IMPACT) and recruitment details have been described elsewhere [see ^15, 16^]. Briefly, patients fulfilled DSM-IV-TR criteria for ADHD in childhood, as well as adulthood (as determined using the Diagnostic Interview for Adult ADHD ^17^). Exclusion criteria for participants included psychosis, alcohol or substance addiction in the last 6 months, current major depression (assessed using SCID-I), full-scale IQ estimate <70 (assessed using the Wechsler Adult Intelligence Scale-III), neurological disorders, sensorimotor disabilities, non-Caucasian ethnicity, and medication use other than psychostimulants or atomoxetine. Controls matched for age, gender, and IQ were recruited from the department of Psychiatry of the Radboud University Nijmegen Medical Centre (Nijmegen, the Netherlands). An additional exclusion criterion for healthy comparison subjects was a current or past neurological or psychiatric disorder according to SCID-I. |
| VII | HC | This cohort has been described in detail elsewhere [see ^18^]. In short, controls were recruited from the Based area in Switzerland. Exclusion criteria included the presence of neurological or psychiatric illness or use of medication (other than oral contraceptives) at the time of recruitment. |

**Supplementary Table 3. VBM-based features used in the present study.** P indicates the univariate P-value (P_FDR_ for FDR adjusted P-values) determined from cohorts I to IV). Features with P_FDR_ < 0.05 are shown in bold. t represents the corresponding t-statistic (negative values: decrease in patients). VE: Percentage of variance explained by a random forest model built on 22 global structural parameters (see Supplementary Table 10). P-GC_FDR_ indicates the FDR adjusted P-values after correction against the 22 global structural parameters

| **Variable name** | **P** | **P_FDR_** | **t** | **VE** | **P-GC_FDR_** | **t-GC** |
| --- | --- | --- | --- | --- | --- | --- |
| EstimatedTotalIntraCranialVol | 0.0942 | 0.104 | -1.68 | 95 | 0.895 | 0.62 |
| **Left_Amygdala** | 1.1e-11 | 1.36e-10 | -6.9 | 39 | 0.122 | -2.76 |
| **Left_Angular** | 0.000465 | 0.000657 | -3.52 | 27 | 0.633 | 1.28 |
| **Left_Anterior_Cingulum** | 1.6e-08 | 4.93e-08 | -5.71 | 40 | 0.895 | -0.43 |
| **Left_Calcarine** | 0.00919 | 0.0108 | -2.61 | 22 | 0.895 | 0.54 |
| Left_Caudate | 0.981 | 0.981 | 0.02 | 34 | 0.945 | 0.16 |
| Left_Central_Paracentral_Lobule | 0.768 | 0.781 | 0.3 | 10 | 0.152 | 2.47 |
| **Left_Cerebellum** | 3.05e-09 | 1.07e-08 | -6 | 46 | 0.733 | -1.14 |
| **Left_Cerebellum_10** | 0.000174 | 0.000262 | -3.77 | 11 | 0.769 | -1.03 |
| Left_Cerebellum_3 | 0.121 | 0.132 | -1.55 | 14 | 0.766 | 1.08 |
| **Left_Cerebellum_4.5** | 0.00117 | 0.00159 | -3.26 | 26 | 0.895 | 0.52 |
| **Left_Cerebellum_6** | 1.48e-06 | 3.3e-06 | -4.85 | 35 | 0.852 | -0.79 |
| **Left_Cerebellum_7b** | 2.64e-06 | 5.59e-06 | -4.73 | 31 | 0.895 | -0.48 |
| **Left_Cerebellum_8** | 4.41e-05 | 7.32e-05 | -4.11 | 37 | 0.574 | 1.38 |
| **Left_Cerebellum_9** | 2.6e-05 | 4.57e-05 | -4.23 | 25 | 0.945 | -0.16 |
| **Left_Cingulum** | 0.00345 | 0.00434 | -2.93 | 17 | 0.895 | 0.44 |
| **Left_Cuneus** | 0.0274 | 0.0312 | -2.21 | 27 | 0.214 | 2.26 |
| **Left_Fusiform** | 1.83e-12 | 3.72e-11 | -7.17 | 55 | 0.852 | -0.8 |
| **Left_Heschl** | 0.00445 | 0.00548 | -2.85 | 13 | 0.786 | 0.95 |
| **Left_Hippocampus** | 2.48e-09 | 8.96e-09 | -6.04 | 38 | 0.151 | -2.55 |
| **Left_Inferior_Frontal** | 3.68e-14 | 4.52e-12 | -7.72 | 35 | 0.168 | -2.38 |
| **Left_Inferior_Frontal_Operculum** | 4.98e-10 | 2.34e-09 | -6.3 | 32 | 0.852 | -0.79 |
| **Left_Inferior_Frontal_Orbital** | 5.96e-08 | 1.63e-07 | -5.48 | 37 | 0.93 | -0.22 |
| **Left_Inferior_Occipital** | 2.59e-05 | 4.57e-05 | -4.23 | 23 | 0.895 | -0.34 |
| **Left_Inferior_Parietal** | 1.7e-07 | 4.37e-07 | -5.28 | 29 | 0.895 | -0.37 |
| **Left_Inferior_Temporal** | 2.86e-08 | 8.39e-08 | -5.61 | 45 | 0.914 | -0.28 |
| **Left_Insula** | 2.12e-12 | 3.72e-11 | -7.15 | 53 | 0.433 | -1.73 |
| **Left_Lingual** | 3.38e-08 | 9.68e-08 | -5.58 | 35 | 0.928 | -0.25 |
| **Left_Medial_Frontal_Orbital** | 1.17e-12 | 3.59e-11 | -7.23 | 40 | 0.168 | -2.39 |
| **Left_Middle_Cingulum** | 1.46e-09 | 5.61e-09 | -6.13 | 42 | 0.587 | -1.34 |
| **Left_Middle_Frontal** | 2.29e-09 | 8.55e-09 | -6.05 | 49 | 0.985 | 0.02 |
| **Left_Middle_Frontal_Orbital** | 4.97e-10 | 2.34e-09 | -6.3 | 41 | 0.895 | -0.52 |
| **Left_Middle_Occipital** | 4.53e-05 | 7.43e-05 | -4.1 | 34 | 0.852 | 0.79 |
| **Left_Middle_Temporal** | 4.07e-12 | 6.26e-11 | -7.05 | 54 | 0.895 | -0.55 |
| **Left_Middle_Temporal_Pole** | 6.2e-05 | 9.78e-05 | -4.03 | 24 | 0.911 | 0.29 |
| **Left_Olfactory** | 2.44e-06 | 5.27e-06 | -4.75 | 34 | 0.942 | -0.2 |
| **Left_Pallidum** | 1.77e-10 | 1.09e-09 | 6.48 | 11 | 2.52e-05 | 5.25 |
| **Left_Parahippocampus** | 1.33e-10 | 9.25e-10 | -6.52 | 36 | 0.587 | -1.34 |
| **Left_Postcentral** | 7.4e-06 | 1.52e-05 | -4.51 | 25 | 0.895 | -0.51 |
| **Left_Precentral** | 0.00723 | 0.00872 | -2.69 | 28 | 0.433 | 1.73 |
| **Left_Precuneus** | 0.00149 | 0.00199 | -3.19 | 38 | 0.416 | 1.8 |
| **Left_Putamen** | 0.00385 | 0.00479 | 2.9 | 28 | 0.0854 | 2.95 |
| **Left_Rectus** | 5.58e-11 | 4.9e-10 | -6.65 | 39 | 0.752 | -1.11 |
| **Left_Rolandic_Operculum** | 1.53e-07 | 4.01e-07 | -5.3 | 40 | 0.895 | 0.44 |
| **Left_Superior_Frontal** | 4.66e-07 | 1.1e-06 | -5.09 | 43 | 0.895 | 0.52 |
| **Left_Superior_Frontal_Orbital** | 1.21e-10 | 9.25e-10 | -6.53 | 45 | 0.818 | -0.88 |
| **Left_Superior_Medial_Frontal** | 1.43e-10 | 9.25e-10 | -6.51 | 51 | 0.895 | -0.53 |
| **Left_Superior_Motor** | 0.00194 | 0.00254 | -3.11 | 29 | 0.574 | 1.39 |
| **Left_Superior_Occipital** | 0.00269 | 0.00345 | -3.01 | 18 | 0.772 | 1.01 |
| **Left_Superior_Parietal** | 1.8e-05 | 3.35e-05 | -4.32 | 21 | 0.928 | -0.24 |
| **Left_Superior_Temporal** | 3.59e-10 | 1.92e-09 | -6.36 | 41 | 0.895 | -0.36 |
| **Left_Superior_Temporal_Pole** | 9.19e-08 | 2.46e-07 | -5.4 | 34 | 0.895 | -0.42 |
| **Left_Supramarginal** | 4.33e-10 | 2.22e-09 | -6.33 | 28 | 0.572 | -1.51 |
| **Left_Thalamus** | 5e-05 | 8.05e-05 | -4.08 | 20 | 0.895 | -0.41 |
| **Left_Vermis** | 0.000545 | 0.000753 | -3.47 | 10 | 0.895 | -0.53 |
| Left_Vermis_1.2 | 0.592 | 0.622 | -0.54 | 6 | 0.896 | 0.32 |
| **Left_Vermis_10** | 0.0291 | 0.0328 | -2.19 | 4 | 0.895 | -0.39 |
| Left_Vermis_3 | 0.225 | 0.243 | -1.21 | 7 | 0.895 | 0.56 |
| Left_Vermis_4.5 | 0.116 | 0.128 | -1.57 | 14 | 0.896 | 0.32 |
| **Left_Vermis_6** | 5.04e-05 | 8.05e-05 | -4.08 | 17 | 0.678 | -1.21 |
| **Left_Vermis_8** | 1.42e-05 | 2.82e-05 | -4.37 | 20 | 0.945 | -0.17 |
| **Left_Vermis_9** | 8.78e-09 | 2.92e-08 | -5.82 | 21 | 0.261 | -2.13 |
| **Right_Amygdala** | 1.54e-11 | 1.72e-10 | -6.85 | 38 | 0.0854 | -2.93 |
| **Right_Angular** | 0.000172 | 0.000261 | -3.78 | 32 | 0.786 | 0.92 |
| **Right_Anterior_Cingulum** | 9.18e-05 | 0.000141 | -3.93 | 32 | 0.895 | 0.56 |
| **Right_Calcarine** | 0.000662 | 0.000904 | -3.42 | 22 | 0.895 | -0.36 |
| Right_Caudate | 0.636 | 0.663 | 0.47 | 32 | 0.895 | 0.57 |
| Right_Central_Paracentral_Lobule | 0.497 | 0.527 | 0.68 | 7 | 0.151 | 2.57 |
| **Right_Cerebellum** | 1.47e-05 | 2.86e-05 | -4.36 | 38 | 0.663 | 1.24 |
| **Right_Cerebellum_10** | 6.12e-06 | 1.28e-05 | -4.56 | 11 | 0.437 | -1.69 |
| Right_Cerebellum_3 | 0.819 | 0.826 | -0.23 | 12 | 0.261 | 2.13 |
| **Right_Cerebellum_4.5** | 0.0223 | 0.0259 | -2.29 | 26 | 0.574 | 1.39 |
| **Right_Cerebellum_6** | 1.54e-05 | 2.92e-05 | -4.35 | 34 | 0.985 | -0.02 |
| **Right_Cerebellum_7b** | 1.88e-06 | 4.13e-06 | -4.8 | 26 | 0.769 | -1.02 |
| **Right_Cerebellum_8** | 5.58e-08 | 1.56e-07 | -5.49 | 34 | 0.786 | -0.93 |
| **Right_Cerebellum_9** | 2.4e-05 | 4.41e-05 | -4.25 | 22 | 0.895 | -0.51 |
| **Right_Cingulum** | 0.0019 | 0.00251 | -3.12 | 13 | 0.786 | -0.96 |
| **Right_Cuneus** | 0.00284 | 0.0036 | -3 | 24 | 0.574 | 1.47 |
| **Right_Fusiform** | 3.24e-11 | 3.07e-10 | -6.74 | 49 | 0.983 | 0.07 |
| **Right_Heschl** | 2.82e-05 | 4.82e-05 | -4.21 | 17 | 0.983 | 0.07 |
| **Right_Hippocampus** | 2.12e-11 | 2.17e-10 | -6.8 | 32 | 0.026 | -3.43 |
| **Right_Inferior_Frontal** | 5.59e-07 | 1.27e-06 | -5.05 | 38 | 0.895 | 0.4 |
| **Right_Inferior_Frontal_Operculum** | 9e-10 | 3.69e-09 | -6.21 | 31 | 0.717 | -1.16 |
| **Right_Inferior_Frontal_Orbital** | 4.7e-09 | 1.6e-08 | -5.93 | 43 | 0.895 | -0.66 |
| **Right_Inferior_Occipital** | 0.000347 | 0.000507 | -3.59 | 24 | 0.895 | -0.37 |
| **Right_Inferior_Parietal** | 0.0077 | 0.0092 | -2.67 | 24 | 0.39 | 1.91 |
| **Right_Inferior_Temporal** | 5.32e-10 | 2.34e-09 | -6.29 | 47 | 0.852 | -0.8 |
| **Right_Insula** | 3.89e-13 | 1.59e-11 | -7.39 | 50 | 0.414 | -1.86 |
| **Right_Lingual** | 1.28e-08 | 4.14e-08 | -5.75 | 30 | 0.769 | -1.04 |
| **Right_Medial_Frontal_Orbital** | 7.53e-12 | 1.03e-10 | -6.96 | 38 | 0.151 | -2.51 |
| **Right_Middle_Cingulum** | 2.3e-10 | 1.35e-09 | -6.43 | 43 | 0.416 | -1.79 |
| **Right_Middle_Frontal** | 1.4e-08 | 4.43e-08 | -5.74 | 49 | 0.983 | -0.07 |
| **Right_Middle_Frontal_Orbital** | 1.34e-09 | 5.33e-09 | -6.14 | 41 | 0.874 | -0.75 |
| **Right_Middle_Occipital** | 2.72e-05 | 4.72e-05 | -4.22 | 32 | 0.895 | 0.47 |
| **Right_Middle_Temporal** | 1.47e-12 | 3.61e-11 | -7.2 | 61 | 0.895 | -0.66 |
| **Right_Middle_Temporal_Pole** | 1.79e-07 | 4.5e-07 | -5.27 | 28 | 0.895 | -0.47 |
| **Right_Olfactory** | 9.42e-06 | 1.9e-05 | -4.46 | 35 | 0.985 | -0.05 |
| **Right_Pallidum** | 8.89e-11 | 7.29e-10 | 6.59 | 4 | 0.00015 | 4.75 |
| **Right_Parahippocampus** | 9.32e-14 | 5.73e-12 | -7.6 | 40 | 0.416 | -1.83 |
| **Right_Postcentral** | 0.000421 | 0.000602 | -3.54 | 22 | 0.895 | 0.53 |
| **Right_Precentral** | 2.57e-05 | 4.57e-05 | -4.24 | 28 | 0.985 | -0.03 |
| **Right_Precuneus** | 0.000512 | 0.000715 | -3.49 | 39 | 0.574 | 1.43 |
| **Right_Putamen** | 0.0054 | 0.00658 | 2.79 | 28 | 0.124 | 2.7 |
| **Right_Rectus** | 1.36e-10 | 9.25e-10 | -6.51 | 43 | 0.766 | -1.07 |
| **Right_Rolandic_Operculum** | 7.68e-10 | 3.26e-09 | -6.23 | 43 | 0.93 | -0.23 |
| **Right_Superior_Frontal** | 3.05e-05 | 5.14e-05 | -4.2 | 36 | 0.786 | 0.97 |
| **Right_Superior_Frontal_Orbital** | 2.35e-07 | 5.77e-07 | -5.22 | 45 | 0.895 | 0.56 |
| **Right_Superior_Medial_Frontal** | 5.14e-10 | 2.34e-09 | -6.3 | 33 | 0.437 | -1.68 |
| **Right_Superior_Motor** | 0.0021 | 0.00271 | -3.09 | 32 | 0.478 | 1.62 |
| **Right_Superior_Occipital** | 0.000284 | 0.000421 | -3.65 | 19 | 0.985 | 0.05 |
| **Right_Superior_Parietal** | 0.0373 | 0.0417 | -2.09 | 18 | 0.574 | 1.43 |
| **Right_Superior_Temporal** | 3.38e-10 | 1.89e-09 | -6.37 | 47 | 0.945 | 0.15 |
| **Right_Superior_Temporal_Pole** | 2.06e-08 | 6.19e-08 | -5.67 | 37 | 0.895 | -0.34 |
| **Right_Supramarginal** | 5.16e-07 | 1.2e-06 | -5.06 | 32 | 0.945 | 0.15 |
| **Right_Thalamus** | 0.00781 | 0.00923 | -2.67 | 21 | 0.895 | 0.67 |
| **Right_Vermis** | 6.29e-05 | 9.79e-05 | -4.02 | 7 | 0.574 | -1.4 |
| Right_Vermis_1.2 | 0.68 | 0.697 | -0.41 | 2 | 0.895 | 0.47 |
| **Right_Vermis_10** | 0.0257 | 0.0295 | -2.24 | 5 | 0.895 | -0.57 |
| Right_Vermis_3 | 0.662 | 0.685 | 0.44 | 5 | 0.323 | 2.01 |
| Right_Vermis_4.5 | 0.302 | 0.323 | -1.03 | 12 | 0.786 | 0.93 |
| **Right_Vermis_6** | 0.000383 | 0.000555 | -3.57 | 15 | 0.856 | -0.77 |
| **Right_Vermis_8** | 1.49e-05 | 2.87e-05 | -4.36 | 20 | 0.895 | -0.43 |
| **Right_Vermis_9** | 2.84e-07 | 6.85e-07 | -5.18 | 19 | 0.574 | -1.38 |

**Supplementary Table 4. FreeSurfer-based features used in the present study.** P indicates the univariate P-value (P_FRD_ for FDR adjusted P-values) determined from cohorts I to IV). Features with P_FDR_ < 0.05 are shown in bold. t represents the corresponding t-statistic (negative values: decrease in patients). VE: Percentage of variance explained by a random forest model built on 22 global structural parameters (see Supplementary Table 10).

| **Variable name** | **P** | **P_FDR_** | **t** | **VE** | **P-GC_FDR_** | **t-GC** |
| --- | --- | --- | --- | --- | --- | --- |
| CC_Anterior | 0.0891 | 0.117 | -1.7 | 12 | 0.973 | 0.18 |
| **CC_Central** | 1.16e-05 | 5.38e-05 | -4.42 | 33 | 0.832 | -0.89 |
| **CC_Mid_Anterior** | 1.86e-05 | 7.51e-05 | -4.31 | 30 | 0.794 | -1.41 |
| **CC_Mid_Posterior** | 1.1e-05 | 5.38e-05 | -4.43 | 31 | 0.85 | -0.71 |
| **CC_Posterior** | 0.00152 | 0.00353 | -3.18 | 14 | 0.653 | -1.58 |
| EstimatedTotalIntraCranialVol | 0.0942 | 0.123 | -1.68 | 95 | 0.844 | 0.78 |
| **Left_Accumbens_area** | 0.0179 | 0.0299 | -2.37 | 11 | 0.872 | -0.65 |
| **Left_Amygdala** | 1.52e-05 | 6.28e-05 | -4.36 | 26 | 0.377 | -2.07 |
| Left_Caudate | 0.0957 | 0.124 | 1.67 | 28 | 0.925 | 0.39 |
| **Left_Cerebellum_Cortex** | 1.08e-06 | 7.85e-06 | -4.92 | 62 | 0.794 | -1.26 |
| **Left_Hippocampus** | 1.34e-06 | 9.32e-06 | -4.88 | 26 | 0.377 | -2.06 |
| **Left_Pallidum** | 0.000462 | 0.00124 | 3.52 | 16 | 0.832 | 0.98 |
| **Left_Putamen** | 0.0291 | 0.0424 | 2.19 | 33 | 0.832 | 1.06 |
| Left_Thalamus_Proper | 0.106 | 0.136 | -1.62 | 33 | 0.832 | 0.96 |
| lh_bankssts_area | 0.162 | 0.196 | -1.4 | 13 | 0.939 | 0.29 |
| lh_caudalanteriorcingulate_area | 0.306 | 0.339 | -1.02 | 10 | 0.872 | 0.58 |
| lh_caudalanteriorcingulate_thickness | 0.213 | 0.249 | -1.25 | 8 | 0.872 | 0.54 |
| lh_caudalmiddlefrontal_area | 0.222 | 0.258 | -1.22 | 12 | 0.832 | 0.93 |
| **lh_caudalmiddlefrontal_thickness** | 4.94e-08 | 4.72e-07 | -5.51 | 52 | 0.794 | -1.32 |
| **lh_cuneus_area** | 0.0024 | 0.00513 | -3.05 | 20 | 0.872 | -0.55 |
| lh_cuneus_thickness | 0.918 | 0.928 | -0.1 | 25 | 0.794 | 1.39 |
| lh_frontalpole_area | 0.634 | 0.665 | -0.48 | 3 | 0.993 | -0.08 |
| **lh_frontalpole_thickness** | 0.00242 | 0.00513 | -3.04 | 7 | 0.832 | -0.87 |
| **lh_fusiform_area** | 0.00134 | 0.0032 | -3.22 | 38 | 0.936 | -0.33 |
| **lh_fusiform_thickness** | 6.2e-09 | 9.48e-08 | -5.88 | 45 | 0.872 | 0.6 |
| **lh_inferiorparietal_area** | 0.00401 | 0.00797 | -2.89 | 25 | 0.993 | -0.12 |
| **lh_inferiorparietal_thickness** | 8.48e-05 | 0.00027 | -3.95 | 55 | 0.794 | 1.24 |
| **lh_inferiortemporal_area** | 0.00368 | 0.0074 | -2.91 | 28 | 0.872 | -0.59 |
| **lh_inferiortemporal_thickness** | 5.31e-07 | 4.06e-06 | -5.06 | 48 | 0.832 | 0.9 |
| lh_insula_area | 0.692 | 0.715 | -0.4 | 12 | 0.825 | 1.17 |
| **lh_insula_thickness** | 9.47e-09 | 1.32e-07 | -5.81 | 37 | 0.832 | -1.11 |
| lh_isthmuscingulate_area | 0.915 | 0.928 | 0.11 | 16 | 0.925 | 0.42 |
| **lh_isthmuscingulate_thickness** | 0.000939 | 0.00236 | -3.32 | 9 | 0.993 | 0.09 |
| lh_lateraloccipital_area | 0.0673 | 0.0903 | -1.83 | 27 | 0.939 | 0.28 |
| lh_lateraloccipital_thickness | 0.161 | 0.196 | -1.4 | 43 | 0.377 | 2.08 |
| **lh_lateralorbitofrontal_area** | 2e-05 | 7.86e-05 | -4.29 | 46 | 0.844 | -0.78 |
| **lh_lateralorbitofrontal_thickness** | 2.58e-06 | 1.62e-05 | -4.74 | 30 | 0.568 | -1.74 |
| **lh_lingual_area** | 0.00112 | 0.00275 | -3.27 | 32 | 0.872 | -0.66 |
| **lh_lingual_thickness** | 0.00155 | 0.00353 | -3.18 | 27 | 0.872 | -0.5 |
| **lh_medialorbitofrontal_area** | 0.0208 | 0.0339 | -2.32 | 28 | 0.96 | -0.22 |
| **lh_medialorbitofrontal_thickness** | 0.000525 | 0.00138 | -3.48 | 19 | 0.377 | -2.08 |
| **lh_middletemporal_area** | 0.000744 | 0.0019 | -3.39 | 33 | 0.794 | -1.26 |
| **lh_middletemporal_thickness** | 3.88e-11 | 5.93e-09 | -6.71 | 51 | 0.872 | -0.5 |
| lh_paracentral_area | 0.924 | 0.928 | 0.1 | 5 | 0.832 | 0.83 |
| **lh_paracentral_thickness** | 0.027 | 0.0405 | -2.22 | 37 | 0.519 | 1.85 |
| **lh_parahippocampal_area** | 0.0273 | 0.0405 | -2.21 | 19 | 0.872 | -0.67 |
| **lh_parahippocampal_thickness** | 3.4e-06 | 1.86e-05 | -4.68 | 19 | 0.85 | -0.71 |
| **lh_parsopercularis_area** | 0.0159 | 0.0275 | -2.42 | 11 | 0.872 | -0.52 |
| **lh_parsopercularis_thickness** | 3.36e-08 | 3.92e-07 | -5.58 | 47 | 0.794 | -1.22 |
| **lh_parsorbitalis_area** | 0.018 | 0.0299 | -2.37 | 24 | 0.994 | -0.03 |
| **lh_parsorbitalis_thickness** | 4.41e-08 | 4.5e-07 | -5.53 | 25 | 0.519 | -1.83 |
| **lh_parstriangularis_area** | 0.0191 | 0.0314 | -2.35 | 14 | 0.872 | -0.5 |
| **lh_parstriangularis_thickness** | 1.41e-06 | 9.4e-06 | -4.86 | 36 | 0.377 | -2.13 |
| **lh_pericalcarine_area** | 0.00144 | 0.00339 | -3.2 | 15 | 0.832 | -0.84 |
| lh_pericalcarine_thickness | 0.174 | 0.207 | 1.36 | 26 | 0.832 | 0.9 |
| lh_postcentral_area | 0.172 | 0.206 | -1.37 | 32 | 0.994 | 0.06 |
| **lh_postcentral_thickness** | 0.00357 | 0.00729 | -2.92 | 48 | 0.85 | 0.71 |
| **lh_posteriorcingulate_area** | 0.0232 | 0.0373 | -2.28 | 13 | 0.872 | -0.56 |
| **lh_posteriorcingulate_thickness** | 0.000462 | 0.00124 | -3.52 | 21 | 0.993 | -0.11 |
| lh_precentral_area | 0.123 | 0.155 | -1.54 | 27 | 0.947 | 0.26 |
| **lh_precentral_thickness** | 3.06e-05 | 0.000115 | -4.19 | 55 | 0.832 | 1 |
| lh_precuneus_area | 0.112 | 0.143 | -1.59 | 40 | 0.85 | 0.72 |
| **lh_precuneus_thickness** | 0.000199 | 0.000565 | -3.74 | 57 | 0.832 | 0.97 |
| lh_rostralanteriorcingulate_area | 0.0439 | 0.0616 | -2.02 | 24 | 0.872 | 0.49 |
| **lh_rostralanteriorcingulate_thickness** | 0.0285 | 0.042 | -2.19 | 8 | 0.967 | 0.19 |
| **lh_rostralmiddlefrontal_area** | 0.00319 | 0.00669 | -2.96 | 46 | 0.925 | 0.4 |
| **lh_rostralmiddlefrontal_thickness** | 3.67e-09 | 7.02e-08 | -5.97 | 54 | 0.452 | -1.94 |
| lh_superiorfrontal_area | 0.0461 | 0.0636 | -2 | 42 | 0.832 | 0.95 |
| **lh_superiorfrontal_thickness** | 2.99e-10 | 1.05e-08 | -6.39 | 65 | 0.552 | -1.78 |
| **lh_superiorparietal_area** | 0.00211 | 0.00467 | -3.09 | 33 | 0.825 | -1.17 |
| lh_superiorparietal_thickness | 0.0862 | 0.115 | -1.72 | 48 | 0.377 | 2.12 |
| **lh_superiortemporal_area** | 0.0251 | 0.0388 | -2.24 | 38 | 0.967 | 0.2 |
| **lh_superiortemporal_thickness** | 3.44e-10 | 1.05e-08 | -6.36 | 48 | 0.872 | -0.49 |
| lh_supramarginal_area | 0.317 | 0.348 | -1 | 24 | 0.832 | 0.92 |
| **lh_supramarginal_thickness** | 1.13e-07 | 9.59e-07 | -5.36 | 60 | 0.939 | -0.28 |
| lh_temporalpole_area | 0.352 | 0.382 | -0.93 | 8 | 0.993 | -0.13 |
| **lh_temporalpole_thickness** | 0.000101 | 0.000309 | -3.91 | 22 | 0.994 | 0.01 |
| lh_transversetemporal_area | 0.149 | 0.182 | -1.44 | 14 | 0.925 | 0.37 |
| **lh_transversetemporal_thickness** | 0.00604 | 0.0116 | -2.75 | 16 | 0.872 | 0.55 |
| rh_bankssts_area | 0.237 | 0.273 | -1.18 | 17 | 0.872 | 0.62 |
| **rh_bankssts_thickness** | 5.72e-05 | 0.000194 | -4.05 | 36 | 0.832 | 0.89 |
| rh_caudalanteriorcingulate_area | 0.662 | 0.689 | 0.44 | 3 | 0.776 | 1.47 |
| rh_caudalanteriorcingulate_thickness | 0.138 | 0.171 | -1.49 | 8 | 0.925 | 0.4 |
| **rh_caudalmiddlefrontal_area** | 0.00861 | 0.0157 | -2.63 | 13 | 0.872 | -0.62 |
| **rh_caudalmiddlefrontal_thickness** | 4.21e-05 | 0.00015 | -4.12 | 49 | 0.993 | 0.09 |
| **rh_cuneus_area** | 0.0117 | 0.0206 | -2.53 | 23 | 0.994 | 0.02 |
| rh_cuneus_thickness | 0.753 | 0.773 | -0.32 | 25 | 0.832 | 0.97 |
| rh_frontalpole_area | 0.0444 | 0.0618 | -2.01 | -2 | 0.794 | -1.35 |
| rh_frontalpole_thickness | 0.0389 | 0.0558 | -2.07 | 9 | 0.993 | -0.12 |
| **rh_fusiform_area** | 0.0022 | 0.00481 | -3.07 | 37 | 0.925 | -0.36 |
| **rh_fusiform_thickness** | 2.51e-10 | 1.05e-08 | -6.42 | 43 | 0.939 | -0.29 |
| **rh_inferiorparietal_area** | 3.09e-05 | 0.000115 | -4.19 | 27 | 0.318 | -2.32 |
| **rh_inferiorparietal_thickness** | 0.000159 | 0.000468 | -3.8 | 56 | 0.318 | 2.35 |
| **rh_inferiortemporal_area** | 0.00904 | 0.0163 | -2.62 | 29 | 0.872 | -0.6 |
| **rh_inferiortemporal_thickness** | 2.77e-06 | 1.62e-05 | -4.72 | 41 | 0.832 | 1.06 |
| **rh_insula_area** | 0.016 | 0.0275 | -2.41 | 10 | 0.85 | -0.74 |
| **rh_insula_thickness** | 2.65e-06 | 1.62e-05 | -4.73 | 27 | 0.872 | -0.58 |
| rh_isthmuscingulate_area | 0.377 | 0.401 | -0.88 | 21 | 0.872 | -0.52 |
| **rh_isthmuscingulate_thickness** | 1.3e-05 | 5.69e-05 | -4.39 | 6 | 0.832 | -1.09 |
| **rh_lateraloccipital_area** | 0.0268 | 0.0405 | -2.22 | 25 | 0.993 | -0.11 |
| rh_lateraloccipital_thickness | 0.0593 | 0.0803 | -1.89 | 41 | 0.318 | 2.47 |
| rh_lateralorbitofrontal_area | 0.0393 | 0.0558 | -2.06 | 36 | 0.832 | 0.93 |
| **rh_lateralorbitofrontal_thickness** | 1.15e-09 | 2.52e-08 | -6.17 | 33 | 0.318 | -2.78 |
| **rh_lingual_area** | 8.47e-06 | 4.32e-05 | -4.49 | 40 | 0.603 | -1.67 |
| **rh_lingual_thickness** | 0.00113 | 0.00275 | -3.27 | 25 | 0.794 | -1.29 |
| **rh_medialorbitofrontal_area** | 0.00694 | 0.0131 | -2.71 | 29 | 0.925 | 0.35 |
| **rh_medialorbitofrontal_thickness** | 8.04e-05 | 0.000262 | -3.97 | 16 | 0.318 | -2.64 |
| **rh_middletemporal_area** | 0.00719 | 0.0132 | -2.7 | 42 | 0.96 | 0.23 |
| **rh_middletemporal_thickness** | 2.86e-07 | 2.31e-06 | -5.18 | 50 | 0.832 | 1.04 |
| rh_paracentral_area | 0.928 | 0.928 | 0.09 | 5 | 0.925 | 0.36 |
| **rh_paracentral_thickness** | 0.00597 | 0.0116 | -2.76 | 36 | 0.625 | 1.64 |
| rh_parahippocampal_area | 0.0394 | 0.0558 | -2.06 | 29 | 0.994 | 0.01 |
| **rh_parahippocampal_thickness** | 0.000126 | 0.000378 | -3.86 | 18 | 0.939 | -0.32 |
| rh_parsopercularis_area | 0.263 | 0.296 | -1.12 | 11 | 0.832 | 1.13 |
| **rh_parsopercularis_thickness** | 1.2e-05 | 5.38e-05 | -4.41 | 39 | 0.832 | -0.92 |
| **rh_parsorbitalis_area** | 0.000704 | 0.00182 | -3.4 | 25 | 0.794 | -1.26 |
| **rh_parsorbitalis_thickness** | 9.26e-08 | 8.34e-07 | -5.39 | 25 | 0.318 | -2.37 |
| **rh_parstriangularis_area** | 0.027 | 0.0405 | -2.22 | 8 | 0.994 | -0.06 |
| **rh_parstriangularis_thickness** | 6.08e-06 | 3.21e-05 | -4.56 | 39 | 0.832 | -0.84 |
| **rh_pericalcarine_area** | 0.00346 | 0.00716 | -2.93 | 18 | 0.872 | -0.63 |
| rh_pericalcarine_thickness | 0.21 | 0.248 | 1.25 | 19 | 0.832 | 0.86 |
| rh_postcentral_area | 0.239 | 0.273 | -1.18 | 31 | 0.947 | 0.26 |
| **rh_postcentral_thickness** | 0.000198 | 0.000565 | -3.74 | 40 | 0.993 | -0.15 |
| rh_posteriorcingulate_area | 0.0498 | 0.068 | -1.97 | 16 | 0.96 | -0.22 |
| **rh_posteriorcingulate_thickness** | 6.68e-05 | 0.000222 | -4.01 | 19 | 0.832 | -0.99 |
| **rh_precentral_area** | 0.0236 | 0.0375 | -2.27 | 30 | 0.872 | -0.64 |
| **rh_precentral_thickness** | 9.18e-05 | 0.000287 | -3.93 | 51 | 0.794 | 1.22 |
| rh_precuneus_area | 0.323 | 0.353 | -0.99 | 35 | 0.603 | 1.69 |
| **rh_precuneus_thickness** | 4.6e-05 | 0.00016 | -4.1 | 48 | 0.794 | 1.26 |
| rh_rostralanteriorcingulate_area | 0.248 | 0.281 | -1.16 | 14 | 0.832 | 0.98 |
| rh_rostralanteriorcingulate_thickness | 0.361 | 0.389 | -0.91 | 15 | 0.85 | 0.72 |
| **rh_rostralmiddlefrontal_area** | 0.0245 | 0.0382 | -2.25 | 41 | 0.832 | 0.93 |
| **rh_rostralmiddlefrontal_thickness** | 4.36e-09 | 7.41e-08 | -5.94 | 50 | 0.318 | -2.32 |
| rh_superiorfrontal_area | 0.122 | 0.154 | -1.55 | 42 | 0.794 | 1.34 |
| **rh_superiorfrontal_thickness** | 6.77e-10 | 1.73e-08 | -6.25 | 62 | 0.435 | -1.98 |
| **rh_superiorparietal_area** | 0.00713 | 0.0132 | -2.7 | 35 | 0.939 | -0.31 |
| **rh_superiorparietal_thickness** | 0.0245 | 0.0382 | -2.25 | 45 | 0.653 | 1.59 |
| **rh_superiortemporal_area** | 0.00965 | 0.0172 | -2.6 | 51 | 0.994 | 0.04 |
| **rh_superiortemporal_thickness** | 1.16e-10 | 8.9e-09 | -6.54 | 55 | 0.925 | -0.35 |
| rh_supramarginal_area | 0.377 | 0.401 | -0.88 | 19 | 0.794 | 1.29 |
| **rh_supramarginal_thickness** | 3.58e-08 | 3.92e-07 | -5.57 | 55 | 0.872 | -0.61 |
| rh_temporalpole_area | 0.294 | 0.328 | -1.05 | 1 | 0.844 | -0.8 |
| **rh_temporalpole_thickness** | 2.85e-06 | 1.62e-05 | -4.72 | 20 | 0.832 | -0.85 |
| rh_transversetemporal_area | 0.386 | 0.407 | -0.87 | 12 | 0.844 | 0.78 |
| **rh_transversetemporal_thickness** | 0.000324 | 0.000901 | -3.61 | 19 | 0.994 | 0.01 |
| **Right_Accumbens_area** | 1.19e-05 | 5.38e-05 | -4.41 | 20 | 0.794 | -1.33 |
| **Right_Amygdala** | 0.00496 | 0.00972 | -2.82 | 25 | 0.85 | -0.75 |
| Right_Caudate | 0.144 | 0.177 | 1.46 | 28 | 0.994 | 0.03 |
| **Right_Cerebellum_Cortex** | 3.67e-05 | 0.000134 | -4.15 | 61 | 0.832 | 1.14 |
| **Right_Hippocampus** | 3.46e-08 | 3.92e-07 | -5.58 | 30 | 0.318 | -2.47 |
| **Right_Pallidum** | 1.36e-05 | 5.78e-05 | 4.38 | 26 | 0.143 | 3.32 |
| **Right_Putamen** | 0.00193 | 0.00435 | 3.11 | 37 | 0.318 | 2.71 |
| **Right_Thalamus_Proper** | 0.0179 | 0.0299 | -2.37 | 36 | 0.925 | 0.36 |

**Supplementary Table 5. Classification accuracy for within cohort classification of schizophrenia patients and controls.** AUC values were determined from the out-of-bag predictions during random forest classification. Significance estimates for random forest classification were derived from permutation. No significance estimates are shown for SVM computations, due to the large computational load of performing nested cross-validation repeatedly during permutation.

| **Cohort** | **1** | **2** | **3** | **4** |
| --- | --- | --- | --- | --- |
| **Random forest** |  |  |  |  |
| VBM-based | 0.58 (*P* = 0.396) | 0.82 (*P* < 0.001) | 0.61 (*P* = 0.025) | 0.74 (*P* = 0.004) |
| FreeSurfer-based | 0.58 (*P* = 0.114) | 0.80 (*P* < 0.001) | 0.64 (*P* = 0.004) | 0.73 (*P* = 0.005) |
| **SVM** |  |  |  |  |
| VBM-based | 0.62 | 0.82 | 0.85 | 0.77 |
| FreeSurfer-based | 0.64 | 0.80 | 0.90 | 0.68 |

**Supplementary Table 6. Classification accuracy for leave-site-out classification of schizophrenia patients and controls, using no test data for building of normalization models.**

| **Cohort** | **I** | **II** | **III** | **IV** |
| --- | --- | --- | --- | --- |
| **Random forest** |  |  |  |  |
| VBM-based |  |  |  |  |
| AUC | 0.70 | 0.83 | 0.90 | 0.63 |
| Sensitivity/Specificity | 53/77 | 69/80 | 87/72 | 88/23 |
| FreeSurfer-based |  |  |  |  |
| AUC | 0.54 | 0.66 | 0.78 | 0.61 |
| Sensitivity/Specificity | 2/100 | 5/99 | 100/61 | 98/0 |
| **SVM** |  |  |  |  |
| VBM-based |  |  |  |  |
| AUC | 0.71 | 0.86 | 0.91 | 0.77 |
| Sensitivity/Specificity | 49/77 | 69/88 | 90/73 | 91/18 |
| FreeSurfer-based |  |  |  |  |
| AUC | 0.66 | 0.74 | 0.88 | 0.72 |
| Sensitivity/Specificity | 0/100 | 29/97 | 99/62 | 100/0 |

**Supplementary Table 7. Classification accuracy for leave-site-out classification of schizophrenia patients and controls, after setting the mean of each structural feature in the test data to zero.**

| **Cohort** | **I** | **II** | **III** | **IV** |
| --- | --- | --- | --- | --- |
| **Random forest** |  |  |  |  |
| VBM-based |  |  |  |  |
| AUC | 0.70 | 0.84 | 0.90 | 0.74 |
| Sensitivity/Specificity | 61/75 | 73/84 | 87/73 | 68/79 |
| FreeSurfer-based |  |  |  |  |
| AUC | 0.64 | 0.80 | 0.88 | 0.69 |
| Sensitivity/Specificity | 63/59 | 80/71 | 99/63 | 61/73 |
| **SVM** |  |  |  |  |
| VBM-based |  |  |  |  |
| AUC | 0.71 | 0.85 | 0.91 | 0.77 |
| Sensitivity/Specificity | 67/70 | 76/76 | 87/78 | 70/80 |
| FreeSurfer-based |  |  |  |  |
| AUC | 0.66 | 0.74 | 0.88 | 0.72 |
| Sensitivity/Specificity | 65/57 | 76/57 | 95/67 | 61/77 |

**Supplementary Table 8. Specificity testing using validation cohorts. Values show the obtained specificity estimates in per cent.**

| **Cohort** | **V_HC_** | **VI_HC_** | **VII_HC_** | **VIII_HC_** | **VIII_BD_** | **V_ADHD_** | **VI_ADHD_** |
| --- | --- | --- | --- | --- | --- | --- | --- |
| **Random forest** |  |  |  |  |  |  |  |
| VBM-based | 76 | 89 | 59 | 49 | 31 | 77 | 87 |
| FreeSurfer-based | 52 | 23 | 72 | 44 | 33 | 45 | 16 |
| Combined | 60 | 89 | 65 | 51 | 28 | 75 | 82 |
| **SVM** |  |  |  |  |  |  |  |
| VBM-based | 75 | 86 | 66 | 56 | 55 | 67 | 87 |
| FreeSurfer-based | 35 | 20 | 61 | 55 | 48 | 25 | 15 |
| Combined | 52 | 84 | 65 | 59 | 50 | 56 | 84 |

**Supplementary Table 9. Ranked variable importance determined from random forest classification for VBM-based features.** The 14 features marked with * were selected as the most important predictors by random forest variable selection.

| **Rank** | **Variable name** |
| --- | --- |
| 1* | Right_Pallidum |
| 2* | Left_Pallidum |
| 3* | Left_Inferior_Frontal |
| 4* | Right_Rolandic_Operculum |
| 5* | Right_Hippocampus |
| 6 | Left_Amygdala |
| 7* | Left_Fusiform |
| 8 | Left_Inferior_Frontal_Operculum |
| 9 | Right_Cerebellum_8 |
| 10* | Left_Vermis_9 |
| 11* | Right_Parahippocampus |
| 12 | Left_Parahippocampus |
| 13* | Right_Superior_Medial_Frontal |
| 14 | Left_Inferior_Frontal_Orbital |
| 15 | Left_Cerebellum_10 |
| 16 | Right_Fusiform |
| 17* | Right_Amygdala |
| 18 | Right_Middle_Cingulum |
| 19 | Left_Central_Paracentral_Lobule |
| 20 | Left_Hippocampus |
| 21* | Left_Superior_Frontal_Orbital |
| 22* | Left_Middle_Temporal |
| 23 | Left_Superior_Temporal |
| 24 | Left_Middle_Frontal_Orbital |
| 25* | Right_Medial_Frontal_Orbital |
| 26 | Left_Supramarginal |
| 27 | Right_Cerebellum_10 |
| 28 | Left_Middle_Cingulum |
| 29 | Right_Middle_Frontal_Orbital |
| 30 | Left_Insula |
| 31* | Right_Insula |
| 32 | EstimatedTotalIntraCranialVol |
| 33 | Right_Central_Paracentral_Lobule |
| 34 | Left_Superior_Medial_Frontal |
| 35 | Right_Lingual |
| 36 | Left_Cerebellum_6 |
| 37 | Right_Rectus |
| 38 | Left_Putamen |
| 39 | Right_Vermis |
| 40 | Left_Vermis |
| 41 | Right_Middle_Temporal |
| 42 | Left_Caudate |
| 43 | Left_Rectus |
| 44 | Right_Inferior_Occipital |
| 45 | Left_Cerebellum_7b |
| 46 | Right_Putamen |
| 47 | Right_Superior_Temporal |
| 48 | Right_Cerebellum_3 |
| 49 | Left_Cerebellum_9 |
| 50 | Right_Cerebellum_6 |
| 51 | Right_Cerebellum_7b |
| 52 | Left_Cerebellum |
| 53 | Right_Vermis_9 |
| 54 | Right_Calcarine |
| 55 | Right_Inferior_Frontal_Orbital |
| 56 | Left_Cingulum |
| 57 | Left_Inferior_Parietal |
| 58 | Left_Olfactory |
| 59 | Left_Superior_Temporal_Pole |
| 60 | Right_Caudate |
| 61 | Right_Inferior_Frontal_Operculum |
| 62 | Right_Superior_Temporal_Pole |
| 63 | Right_Heschl |
| 64 | Left_Anterior_Cingulum |
| 65 | Left_Postcentral |
| 66 | Left_Inferior_Occipital |
| 67 | Left_Vermis_8 |
| 68 | Right_Inferior_Temporal |
| 69 | Right_Supramarginal |
| 70 | Right_Cuneus |
| 71 | Left_Vermis_10 |
| 72 | Right_Precentral |
| 73 | Left_Cerebellum_3 |
| 74 | Right_Vermis_10 |
| 75 | Left_Rolandic_Operculum |
| 76 | Left_Cerebellum_8 |
| 77 | Left_Vermis_6 |
| 78 | Left_Superior_Parietal |
| 79 | Right_Cerebellum_9 |
| 80 | Right_Middle_Frontal |
| 81 | Right_Cerebellum_4.5 |
| 82 | Left_Thalamus |
| 83 | Right_Superior_Motor |
| 84 | Right_Vermis_8 |
| 85 | Right_Olfactory |
| 86 | Right_Anterior_Cingulum |
| 87 | Right_Inferior_Frontal |
| 88 | Right_Thalamus |
| 89 | Right_Superior_Frontal_Orbital |
| 90 | Right_Cingulum |
| 91 | Left_Medial_Frontal_Orbital |
| 92 | Left_Precentral |
| 93 | Left_Calcarine |
| 94 | Right_Precuneus |
| 95 | Right_Inferior_Parietal |
| 96 | Left_Cuneus |
| 97 | Left_Superior_Motor |
| 98 | Right_Postcentral |
| 99 | Right_Vermis_3 |
| 100 | Right_Cerebellum |
| 101 | Right_Middle_Temporal_Pole |
| 102 | Right_Vermis_4.5 |
| 103 | Left_Precuneus |
| 104 | Right_Vermis_1.2 |
| 105 | Left_Lingual |
| 106 | Left_Vermis_3 |
| 107 | Right_Angular |
| 108 | Left_Angular |
| 109 | Left_Middle_Frontal |
| 110 | Right_Vermis_6 |
| 111 | Right_Superior_Occipital |
| 112 | Left_Vermis_1.2 |
| 113 | Right_Superior_Frontal |
| 114 | Left_Cerebellum_4.5 |
| 115 | Left_Superior_Frontal |
| 116 | Left_Middle_Temporal_Pole |
| 117 | Left_Vermis_4.5 |
| 118 | Left_Inferior_Temporal |
| 119 | Left_Middle_Occipital |
| 120 | Right_Superior_Parietal |
| 121 | Left_Heschl |
| 122 | Right_Middle_Occipital |
| 123 | Left_Superior_Occipital |

**Supplementary Table 10. Ranked variable importance determined from random forest classification for FreeSurfer-based features.** The 11 features marked with * were selected as the most important predictors by random forest variable selection.

| **Rank** | **Variable name** |
| --- | --- |
| 1* | Right_Hippocampus |
| 2 | Right_Pallidum |
| 3* | Left_Pallidum |
| 4* | lh_middletemporal_thickness |
| 5 | CC_Mid_Anterior |
| 6* | lh_superiortemporal_thickness |
| 7* | rh_lateralorbitofrontal_thickness |
| 8* | CC_Posterior |
| 9 | rh_inferiorparietal_area |
| 10* | rh_superiorfrontal_thickness |
| 11 | lh_lateralorbitofrontal_area |
| 12 | rh_medialorbitofrontal_thickness |
| 13 | lh_parsopercularis_thickness |
| 14* | lh_rostralmiddlefrontal_thickness |
| 15 | Left_Amygdala |
| 16 | lh_parsorbitalis_thickness |
| 17 | lh_parstriangularis_thickness |
| 18 | Left_Cerebellum_Cortex |
| 19 | Right_Putamen |
| 20 | rh_superiortemporal_thickness |
| 21 | rh_lingual_area |
| 22 | rh_isthmuscingulate_thickness |
| 23 | Left_Hippocampus |
| 24 | lh_fusiform_area |
| 25 | lh_inferiorparietal_area |
| 26 | CC_Mid_Posterior |
| 27 | CC_Central |
| 28* | lh_superiorfrontal_thickness |
| 29 | lh_insula_thickness |
| 30 | lh_transversetemporal_thickness |
| 31 | lh_parahippocampal_thickness |
| 32 | Left_Putamen |
| 33 | rh_pericalcarine_area |
| 34 | rh_temporalpole_thickness |
| 35 | lh_parsorbitalis_area |
| 36 | rh_precuneus_thickness |
| 37* | rh_fusiform_thickness |
| 38 | Left_Thalamus_Proper |
| 39 | lh_lateralorbitofrontal_thickness |
| 40 | rh_supramarginal_thickness |
| 41 | rh_parsorbitalis_thickness |
| 42 | EstimatedTotalIntraCranialVol |
| 43* | rh_rostralmiddlefrontal_thickness |
| 44 | Right_Accumbens_area |
| 45 | rh_posteriorcingulate_thickness |
| 46 | lh_supramarginal_thickness |
| 47 | lh_middletemporal_area |
| 48 | lh_frontalpole_thickness |
| 49 | lh_isthmuscingulate_thickness |
| 50 | lh_posteriorcingulate_thickness |
| 51 | Right_Cerebellum_Cortex |
| 52 | lh_temporalpole_thickness |
| 53 | lh_fusiform_thickness |
| 54 | Right_Caudate |
| 55 | rh_insula_area |
| 56 | rh_lateralorbitofrontal_area |
| 57 | rh_fusiform_area |
| 58 | lh_inferiortemporal_thickness |
| 59 | rh_transversetemporal_thickness |
| 60 | rh_inferiortemporal_area |
| 61 | lh_medialorbitofrontal_area |
| 62 | lh_superiorparietal_area |
| 63 | rh_parahippocampal_thickness |
| 64 | lh_medialorbitofrontal_thickness |
| 65 | rh_middletemporal_area |
| 66 | rh_medialorbitofrontal_area |
| 67 | rh_supramarginal_area |
| 68 | lh_superiorparietal_thickness |
| 69 | lh_parahippocampal_area |
| 70 | rh_parsopercularis_thickness |
| 71 | lh_pericalcarine_area |
| 72 | rh_frontalpole_area |
| 73 | lh_posteriorcingulate_area |
| 74 | rh_transversetemporal_area |
| 75 | lh_isthmuscingulate_area |
| 76 | rh_bankssts_area |
| 77 | rh_rostralanteriorcingulate_thickness |
| 78 | rh_cuneus_area |
| 79 | rh_caudalmiddlefrontal_area |
| 80 | rh_bankssts_thickness |
| 81 | rh_insula_thickness |
| 82 | lh_cuneus_area |
| 83 | rh_lateraloccipital_area |
| 84 | lh_bankssts_area |
| 85 | rh_precentral_area |
| 86 | Right_Amygdala |
| 87 | rh_pericalcarine_thickness |
| 88 | Left_Caudate |
| 89 | lh_caudalmiddlefrontal_thickness |
| 90 | lh_inferiortemporal_area |
| 91 | lh_parstriangularis_area |
| 92 | rh_superiortemporal_area |
| 93 | lh_lateraloccipital_area |
| 94 | Left_Accumbens_area |
| 95 | lh_rostralmiddlefrontal_area |
| 96 | rh_parsopercularis_area |
| 97 | lh_lingual_area |
| 98 | rh_temporalpole_area |
| 99 | CC_Anterior |
| 100 | lh_precuneus_thickness |
| 101 | lh_superiorfrontal_area |
| 102 | rh_parstriangularis_thickness |
| 103 | lh_transversetemporal_area |
| 104 | rh_isthmuscingulate_area |
| 105 | lh_pericalcarine_thickness |
| 106 | rh_parsorbitalis_area |
| 107 | rh_caudalanteriorcingulate_area |
| 108 | lh_precentral_area |
| 109 | lh_precentral_thickness |
| 110 | lh_lingual_thickness |
| 111 | lh_rostralanteriorcingulate_area |
| 112 | rh_parstriangularis_area |
| 113 | rh_caudalanteriorcingulate_thickness |
| 114 | rh_postcentral_thickness |
| 115 | rh_lingual_thickness |
| 116 | rh_posteriorcingulate_area |
| 117 | rh_caudalmiddlefrontal_thickness |
| 118 | lh_lateraloccipital_thickness |
| 119 | lh_caudalanteriorcingulate_thickness |
| 120 | rh_paracentral_area |
| 121 | lh_caudalanteriorcingulate_area |
| 122 | lh_parsopercularis_area |
| 123 | rh_superiorparietal_thickness |
| 124 | lh_rostralanteriorcingulate_thickness |
| 125 | rh_inferiortemporal_thickness |
| 126 | rh_rostralmiddlefrontal_area |
| 127 | rh_superiorparietal_area |
| 128 | lh_caudalmiddlefrontal_area |
| 129 | rh_superiorfrontal_area |
| 130 | lh_paracentral_area |
| 131 | rh_precuneus_area |
| 132 | lh_cuneus_thickness |
| 133 | rh_postcentral_area |
| 134 | lh_paracentral_thickness |
| 135 | Right_Thalamus_Proper |
| 136 | rh_paracentral_thickness |
| 137 | rh_parahippocampal_area |
| 138 | rh_middletemporal_thickness |
| 139 | lh_postcentral_thickness |
| 140 | lh_frontalpole_area |
| 141 | lh_inferiorparietal_thickness |
| 142 | lh_insula_area |
| 143 | lh_precuneus_area |
| 144 | rh_lateraloccipital_thickness |
| 145 | lh_superiortemporal_area |
| 146 | rh_precentral_thickness |
| 147 | rh_inferiorparietal_thickness |
| 148 | lh_postcentral_area |
| 149 | lh_supramarginal_area |
| 150 | lh_temporalpole_area |
| 151 | rh_frontalpole_thickness |
| 152 | rh_rostralanteriorcingulate_area |
| 153 | rh_cuneus_thickness |

**Supplementary Table 11. 22 global structural parameters considered in the present study.** Parameters are ordered by the significance (P_VBM_) of association with the first principal component of the top 14 VBM-based features for classification of schizophrenia (see also Figures 3 and 4a). The significance for associations with the first principal component of the top 11 FreeSurfer-based features is shown for comparison (P_FS_).

| **Feature** | **Description** | **P_VBM_** | **P_FS_** | |  |
| --- | --- | --- | --- | --- | --- |
| medFeature | Median corrected feature | 6.26e-281 | | 0.432 | |
| lhCortexVol | Cortex volume, left hemisphere | 6.99e-130 | | 3.52e-48 | |
| TotalGrayVol | Total gray matter volume | 9.09e-128 | | 3.01e-45 | |
| rhCortexVol | Cortex volume, right hemisphere | 5.89e-127 | | 3.78e-45 | |
| SupraTentorialVolNotVent | Supratentorial volume without ventricles | 3.58e-82 | | 2.01e-07 | |
| BrainSegVolNotVent | Brain Segmentation Volume Without Ventricles | 6.09e-81 | | 3.58e-08 | |
| BrainSegVolNotVentSurf | Brain Segmentation Volume Without Ventricles from Surf | 6.23e-81 | | 3.8e-08 | |
| SupraTentorialVol | Supratentorial volume | 5.97e-72 | | 3.4e-06 | |
| BrainSegVol | Brain Segmentation Volume | 7.39e-71 | | 7.32e-07 | |
| BrainSegVol_to_eTIV | Ratio of BrainSegVol to eTIV | 7.8e-69 | | 5.09e-05 | |
| aparc_lh_WhiteSurfArea_area | White matter surface area, left hemisphere | 3.6e-55 | | 0.00311 | |
| aparc_rh_WhiteSurfArea_area | White matter surface area, right hemisphere | 3.98e-52 | | 0.00196 | |
| SubCortGrayVol | Subcortical gray matter volume | 2.29e-39 | | 3.97e-06 | |
| aparc_rh_MeanThickness_thickness | Mean Thickness - right hemisphere | 4.85e-37 | | 1.78e-270 | |
| aparc_lh_MeanThickness_thickness | Mean Thickness - left hemisphere | 9.64e-36 | | 4.87e-286 | |
| MaskVol_to_eTIV | Ratio of MaskVol to eTIV | 3.17e-20 | | 0.513 | |
| MaskVol | Mask Volume | 3.68e-19 | | 0.995 | |
| lhCorticalWhiteMatterVol | Cortical white matter volume, left hemisphere | 1.29e-17 | | 6.00E-06 | |
| rhCorticalWhiteMatterVol | Cortical white matter volume, right hemisphere | 7.46e-17 | | 8.00E-06 | |
| ventricles | Combined ventricle volume | 6.68e-15 | | 1.74e-07 | |
| CSF | Cerebrospinal fluid | 4.97e-05 | | 0.0051 | |
| ICV | Total intracranial volume | 0.455 | | 0.117 | |

**References**

1. Rausch F, Mier D, Eifler S, Esslinger C, Schilling C, Schirmbeck F *et al.* Reduced activation in ventral striatum and ventral tegmental area during probabilistic decision-making in schizophrenia. *Schizophr Res* 2014; **156**(2-3)**:** 143-149.

2. Eisenacher S, Rausch F, Ainser F, Mier D, Veckenstedt R, Schirmbeck F *et al.* Investigation of metamemory functioning in the at-risk mental state for psychosis. *Psychol Med* 2015; **45**(15)**:** 3329-3340.

3. H. Saß HW, M Zaudig. *Diagnostisches und statistisches Manual psychischer Störungen DSM-IV*. Hogrefe: Göttingen, Germany, 2000.

4. Sheehan DV, Lecrubier Y, Sheehan KH, Amorim P, Janavs J, Weiller E *et al.* The Mini-International Neuropsychiatric Interview (M.I.N.I.): the development and validation of a structured diagnostic psychiatric interview for DSM-IV and ICD-10. *J Clin Psychiatry* 1998; **59 Suppl 20:** 22-33;quiz 34-57.

5. Nesvag R, Schaer M, Haukvik UK, Westlye LT, Rimol LM, Lange EH *et al.* Reduced brain cortical folding in schizophrenia revealed in two independent samples. *Schizophr Res* 2014; **152**(2-3)**:** 333-338.

6. Ekholm B, Ekholm A, Adolfsson R, Vares M, Osby U, Sedvall GC *et al.* Evaluation of diagnostic procedures in Swedish patients with schizophrenia and related psychoses. *Nord J Psychiatry* 2005; **59**(6)**:** 457-464.

7. Orhan F, Fatouros-Bergman H, Goiny M, Malmqvist A, Piehl F, Karolinska Schizophrenia Project C *et al.* CSF GABA is reduced in first-episode psychosis and associates to symptom severity. *Mol Psychiatry* 2017.

8. Spitzer RL, Williams, J.B.W., Gibbon, M., First, M.B.. *Structured Clinical Interview for DSM-III-R— Patient Version (SCID-P). Biometrics Research Department. New York State Psychiatric Institute, New York (NY), USA.*, 1988.

9. First M.B. SRL, Gibbon M., Williams J. . *Structured Clinical Interview for DSM-IV Axis I Disorders–Patient Edition (SCID-I/P, Version 2.0)*, 1996.

10. Doan NT, Kaufmann T, Bettella F, Jorgensen KN, Brandt CL, Moberget T *et al.* Distinct multivariate brain morphological patterns and their added predictive value with cognitive and polygenic risk scores in mental disorders. *Neuroimage Clin* 2017; **15:** 719-731.

11. Pergola G, Trizio S, Di Carlo P, Taurisano P, Mancini M, Amoroso N *et al.* Grey matter volume patterns in thalamic nuclei are associated with familial risk for schizophrenia. *Schizophr Res* 2017; **180:** 13-20.

12. Muller UC, Asherson P, Banaschewski T, Buitelaar JK, Ebstein RP, Eisenberg J *et al.* The impact of study design and diagnostic approach in a large multi-centre ADHD study. Part 1: ADHD symptom patterns. *BMC Psychiatry* 2011; **11:** 54.

13. van Rooij D, Hoekstra PJ, Mennes M, von Rhein D, Thissen AJ, Heslenfeld D *et al.* Distinguishing Adolescents With ADHD From Their Unaffected Siblings and Healthy Comparison Subjects by Neural Activation Patterns During Response Inhibition. *Am J Psychiatry* 2015; **172**(7)**:** 674-683.

14. von Rhein D, Mennes M, van Ewijk H, Groenman AP, Zwiers MP, Oosterlaan J *et al.* The NeuroIMAGE study: a prospective phenotypic, cognitive, genetic and MRI study in children with attention-deficit/hyperactivity disorder. Design and descriptives. *Eur Child Adolesc Psychiatry* 2015; **24**(3)**:** 265-281.

15. Hoogman M, Aarts E, Zwiers M, Slaats-Willemse D, Naber M, Onnink M *et al.* Nitric oxide synthase genotype modulation of impulsivity and ventral striatal activity in adult ADHD patients and healthy comparison subjects. *Am J Psychiatry* 2011; **168**(10)**:** 1099-1106.

16. Onnink AM, Zwiers MP, Hoogman M, Mostert JC, Kan CC, Buitelaar J *et al.* Brain alterations in adult ADHD: effects of gender, treatment and comorbid depression. *Eur Neuropsychopharmacol* 2014; **24**(3)**:** 397-409.

17. J. K. Adult ADHD Diagnostic Assess Treat. 2010. Diagnostic Interview for ADHD in Adults 2.0 (DIVA 2.0).

18. Spalek K, Fastenrath M, Ackermann S, Auschra B, Coynel D, Frey J *et al.* Sex-dependent dissociation between emotional appraisal and memory: a large-scale behavioral and fMRI study. *J Neurosci* 2015; **35**(3)**:** 920-935.
